# Integrin α3/α6 and αV are implicated in ADAM15-activated FAK and EGFR signalling pathway individually and promote non-small-cell lung cancer progression

**DOI:** 10.1038/s41419-022-04928-0

**Published:** 2022-05-21

**Authors:** Jieqi Zhou, Anqi Wang, Tingting Cai, Yue Li, Wenwen Du, Yang Zhang, Ruochen Zhang, Weijie Zhang, Jianjie Zhu, Yuanyuan Zeng, Jian-an Huang, Zeyi Liu

**Affiliations:** 1 Department of Pulmonary and Critical Care Medicine, the First Affiliated Hospital of Soochow University, Suzhou, 215006 China; 2 Institute of Respiratory Diseases, Soochow University, Suzhou, 215006 China; 3 Suzhou Key Laboratory for Respiratory Diseases, Suzhou, 215006 China

**Keywords:** Non-small-cell lung cancer, Targeted therapies

## Abstract

Disintegrin-metalloproteinase 15(ADAM15), a member of disintegrin metalloproteinases (ADAMs), plays important roles in various cancer types. However, the underlying ADAM15 functioning in lung cancer is still unclear. In the present study, we find that ADAM15 regulates the epidermal growth factor receptor/focal adhesion kinase (EGFR/FAK) signalling pathway by interactions with integrins. Integrin αV is involved in ADAM15-mediated FAK signalling. Further, we find that ADAM15 and CD151 were co-expressed, and the presence of ADAM15 affected the integrin α3/α6-related EGFR signalling pathway by cooperating with CD151. In addition, we also prove the effect of ADAM15 on proliferation in nude mice. Finally, we show that ADAM15 is a direct target of miR-204-5p by luciferase reporter assays, qRT-PCR and western blot analyses. Our findings provide molecular and cellular evidence that ADAM15 promotes cell proliferation and metastasis in NSCLC, which might provide a potential target for NSCLC treatment.

## Background

Lung cancer is the leading cause of cancer-related death worldwide [1]. Non-small-cell lung cancer (NSCLC) is divided into squamous cell carcinoma, adenocarcinoma and large cell carcinoma, which account for approximately 80% of all lung cancers [2]. Up to date, a great number of therapies have emerged to NSCLC treatment, such as radiotherapy, chemotherapy and immunotherapy. However, the efficacy of these therapies was limited in patients with advanced cancer [3], and the five-year survival rate is still low. Hence, further study on the mechanism of therapy-resistant NSCLC is urgently needed and is beneficial for its future cancer treatment.

Disintegrin-metalloproteinase 15 (ADAM15) is a disintegrin metalloproteinase (ADAM) that belongs to a protease family which consists 40 putative membrane-bound cell surface glycoproteins. ADAMs are typically composed of 800 to 1,200 amino acids with multiple domains, including prodomains, metalloproteinases, disintegrins, cysteine-rich and EGF-like domains and a cytoplasmic C-terminal tail [4]. The multiple domains of ADAMs allow for a variety of functions, including proteolysis, integrin binding and signal transduction. Cytoplasmic C-terminal tails containing Src homology 2 and 3 recognition sequences can interact with different adapter proteins to mediate signal transduction [5]. The metalloproteinase domain of the ADAM family can mediate extracellular matrix protein degradation and ectodomain shedding of growth factors [6]. ADAM15 is a metalloproteinase located on the cell membrane, and is the only ADAM family member containing a consensus arginine-glycine-aspartic acid (RGD) site in its disintegrin domain (metalloproteinase-RGD-disintegrin protein), which can interact with integrins αVβ3 to regulate cell adhesion and motility [7–9]. While it has been shown that high expression of ADAM15 is correlated with poor prognosis of patients with NSCLC, the underlying mechanism of ADAM15 in NSCLC is still unknown.

The tetraspanin CD151 was the first tetraspanin to be identified as a tumour promoter [10]. CD151 has carcinogenic effects and has been identified as a negative prognostic indicator in a variety of cancers [11, 12]. CD151 usually regulates laminin-binding integrins and controls tumour cell invasion and metastasis through their effects on adhesive and signal transduction functions [13]. Our recent study has identified that CD151 regulate EGFR signalling pathway by affecting integrin α3β1 in NSCLC, based on the research on CD151 and its-related genes [14], we found that ADAM15 was significantly correlated with CD151, which aroused our strong interest in exploring the relationship between ADAM15 and CD151.

The purpose of this study was to investigate the role of ADAM15 in NSCLC progression. We showed ADAM15 and CD151 cooperate to regulate the integrin-dependent EGFR/FAK signalling pathway. The protumoural effects of ADAM15 on NSCLC cell lines were further identified in vivo and in vitro.

## Methods

### Cell lines and culture

Human bronchial epithelial cells (BEAS-2B) and the human NSCLC cell lines A549, H1299, H1650, HCC827 (lung adenocarcinoma cell line), H226 (lung squamous cell carcinoma) and H460 (giant-cell lung carcinoma cell line) were purchased from the Cell Bank of the Chinese Academy of Sciences (Shanghai, China) and cultured at 37 °C with 5% CO_2_ in RPMI 1640 medium (HyClone, South Logan, UT, USA) supplemented with 10% foetal bovine serum (Gibco, Carlsbad, CA) and l-glutamine and antibiotics (Invitrogen, Carlsbad, CA, USA).

### NSCLC tissue samples

Paired NSCLC and adjacent noncancerous lung tissue samples (30 of each) were collected with the informed consent of the patients from the First Affiliated Hospital of Soochow University between 2016 and 2018. None of the patients had received radiotherapy or chemotherapy. The tissue samples were snap-frozen and stored in a cryofreezer at −80 °C, and the study was approved by the Academic Advisory Board of Soochow University. The staging and clinical characteristics of the patients are shown in Supplementary Table 1.

### Immunohistochemical assay

Immunohistochemical assay was performed as previously described [15]. Adjacent sections of serial paraffin sections were incubated with anti-CD151 (Santa Cruz, CA, USA, 1:60 dilution in 5% BSA in PBS) and anti-ADAM15 antibodies (Abcam, London, UK, 1:200 dilution in 5% BSA in phosphate-buffered saline (PBS)) at 4 °C overnight and then incubated with the corresponding biotinylated secondary antibodies. The reactions were developed using the DAB Kit (BD Biosciences, San Jose, CA, USA). Detailed protocol was described in the Supplementary materials.

### RNA extraction and quantitative real-time PCR analysis

We used RNAiso Plus (TaKaRa, Osaka, Japan) to extract total RNA from cells and tissues according to the manufacturer’s protocol. We used reverse transcriptase M-MLV (TaKaRa, Osaka, Japan) to synthesize cDNA. Then SYBR Premix Ex TaqTM (TaKaRa, Osaka, Japan) and an ABI Step One Plus Real-Time PCR system (Applied Biosystems, Foster City, CA, USA) were used to perform real-time PCR analysis. Detailed protocol was described in the Supplementary materials.

### RNA interference

A549, H1299 cells were seeded in six-well plates. When the density of cells reached 40–60%, we used Lipofectamine 2000 (Invitrogen) for transfection in accordance with the manufacturer’s instructions. After 48–72 h, the cells were collected for further experiments. The ADAM15, ITGαV, ITGα3 and ITGα6 siRNA and the corresponding controls were purchased from GenePharma Company (Suzhou, China). The target sequences of the siRNAs were described in the Supplementary materials.

### Establishment of stable ADAM15-overexpressing and CD151-overexpressing cell lines

Overexpression lentiviruses for ADAM15 as well as control lentiviruses were purchased from GeneChem Corporation (Shanghai, China), overexpression lentiviruses for CD151 as well as control lentiviruses were purchased from Genomeditech Corporation (Shanghai, China). The cells were then selected with 2 µg/ml puromycin (Sigma-Aldrich, St Louis, MO, USA) to establish a stable cell line for the following experiments.

### Construction of Flag-tagged ADAM15 and HA-tagged CD151 expression vectors

The coding sequences of ADAM15 and CD151 were amplified with the corresponding primers (Supplementary materials) containing Flag tag (for ADAM15) or HA tag (for CD151), and subcloned into a pcDNA3.1 vector to generate pcDNA3.1-ADAM15-Flag or pcDNA3.1-CD151-HA. Before transient transfection, the Flag-tagged ADAM15 or HA-tagged CD151 or sequences of plasmids construct were confirmed by direct sequencing.

### Western blotting assay

Western blotting assays were performed as previously described [16]. The following antibodies were used in the analysis: anti-ADAM15 (sc-365752), anti-CD151 (sc-271216) (Santa Cruz, CA, USA), anti-Integrin αV (ab179475), anti-Integrin α3 (ab242196), anti-Integrin α6 (ab20142), and anti-Integrin β1 (ab52971) antibodies (Abcam, London, UK), followed by the p-Her2 (Sigma-Aldrich; Merck KGaA, SAB4300061), anti-p-EGFR (#3777), anti-EGFR (#4267), anti-Her2 (#4290), anti-p-FAK (#8556), anti-FAK (#13009), anti-p-AKT (#4060), anti-AKT (#4691), anti-p-ERK (#4370), anti-ERK (#4695), anti-CyclinD1 (#2978), anti-MMP9 (#13667) (Cell Signaling Technology Danvers, MA, USA). Anti-β-actin (CW0096M) and anti-mouse (CW0102) or anti-rabbit (CW0103) secondary antibodies were purchased from Cowin (China).

### Co-immunoprecipitation (co-IP) assay

Co-IP assays were performed as previously described [17]. Cell lysates were collected by centrifugation at 10000 × g at 4 °C for 30 min. The supernatants were transferred to a new Eppendorf tube, 1 µg of IgG or the antibody against the target gene was added to each tube and incubated at 4 °C for 24 h with rotation. Then, 50 µl of protein G bead slurry was added to the supernatants, which were incubated at 4 °C for 24 h with rotation. The beads were washed three times with RIPA buffer and then boiled in 2× SDS protein loading buffer for western blot analysis. Detailed protocol was described in the Supplementary materials.

### Cell viability assay

Cell proliferation ability was examined according to the manufacturer’s instructions by a Cell Counting Kit-8 (CCK-8) (Beyotime, Shanghai, China). NSCLC cells were seeded in 96-well plates at a density of 3 × 10^3^ cells per well and further grown in normal culture conditions for 24, 48, and 72 h. A clonogenic assay was also performed to assess cell proliferation. NSCLC cells were seeded in 60-mm plates at a density of 3 × 10^3^ cells/well and incubated for 7–10 days. Colonies formed by at least 50 cells were stained with Giemsa and counted.

### Wound healing assay

NSCLC cells were seeded in 6-well plates, and after 48 hours of transfection, the cell density reached 90%. The monolayer was gently and slowly scratched using a 10 µl pipette tip along with a sterilized ruler, and one horizontal and three vertical scratches were made. Cells were removed by two gentle washes with 1× PBS. The well was replaced with fresh medium, and the cells were cultured for 24 h. Photos of the stained monolayer were taken under a microscope, and the gap distance was quantitatively evaluated using Photoshop.

### Migration and invasion assays

Transwell assays were performed as described previously [16]. For the migration assay, 3 × 10^4^ cells were resuspended in 200 µl of RPMI-1640 medium containing 1% FBS and added into the upper chamber of a Transwell insert, and 800 μl of medium containing 10% FBS was added to the lower chamber. For invasion assays, the inserts were coated with 40 µl Matrigel matrix (BD Science, Sparks, MD, USA) diluted in serum-free medium and incubated at 37 °C for 2 h. Then, the remaining liquid was aspirated from the upper chamber of the insert, 50 µl serum-free medium was added and incubated at 37 °C for 30 min. The remaining liquid was aspirated again, and the remaining procedures were conducted similar to those of the migration assay.

### Cell cycle analysis

The cells were seeded in 6-well plates for 72 hours in RPMI 1640 medium (HyClone, South Logan, UT, USA) supplemented with 5% foetal bovine serum. Then, the cells were washed with PBS, fixed with 70% ethanol at 4 °C overnight, washed with cold PBS again and stained in a propidium iodide (PI)/RNase A mixture according to the instructions of the Cell Cycle Analysis Kit (Beyotime, Shanghai, China). Finally, the cells were kept in the dark at 37 °C for 30 min and tested with a fluorescence-activated cell sorting (FACS) Caliber system (Beckman Coulter, Brea, CA, USA).

### Animal experiments

Female BALB/c athymic nude mice (3–4 weeks old and weighing 16–20 g) were purchased from the Experimental Animal Center of Soochow University and bred under pathogen-free conditions. All animal experiments were carried out in accordance with the Guide for the Care and Use of Experimental Animals Center of Soochow University. Then, A549/vector and A549/ADAM15 cells were suspended in a mixture of RPMI 1640 medium without foetal bovine serum and an equal volume of Matrigel and inoculated subcutaneously into the flanks of nude mice. The control group consisted of 4 mice, and the ADAM15 overexpression group consisted of 12 mice. Then, we randomly divided 8 mice from the ADAM15 overexpression group, where 4 mice were used as the control group, and the other 4 mice were gavaged with the FAK inhibitor defactinib (VS-6063) (Cat. S7654, Selleck, USA) daily. Tumour volume (V) was determined by measuring the length (L) and width (W) with a Vernier calliper and applying the following formula: V = (L × W^2^) × 0.5.

### Immunofluorescence staining

Immunofluorescence staining assays were performed as described previously [18]. Cultured cells were fixed with 4% paraformaldehyde for 15 min at room temperature, washed with PBS every five minutes three times, and blocked with 5% BSA for 1 h at room temperature. Cells were then incubated overnight at 4 °C with anti-ADAM15 (1:100, Santa Cruz) and anti-integrin αV (1:200, Abcam). The corresponding secondary antibodies tagged with Alexa Fluor 647 and FITC were used (1:500, Beyotime Biotechnology). Finally, the samples were incubated in DAPI for 5 min (Life Technologies) for nuclear counterstaining. Images were acquired using a Leica SP8 confocal microscope with optimal settings for the fluorescent markers used.

### Dual-luciferase reporter assay

Dual-luciferase reporter assay were performed as previously described [17]. The luciferase activity was assessed by a Dual-Luciferase Reporter Assay Kit (Promega) and then standardized to the Renilla luciferase activity. Detailed protocol was described in the Supplementary materials.

### Statistical analysis

All data are presented as the mean ± SD. The data of two groups were analysed using an independent-samples t-test. The data of three or more groups were analysed using one-way ANOVA. Differences for which *P* was <0.05 were considered significant. GraphPad Prism 7 software (GraphPad, San Diego, CA, USA) and SPSS 17.0 software were used for statistical analyses.

## Results

### ADAM15 is highly expressed in NSCLC tissues and cell lines

**Fig. 1:**
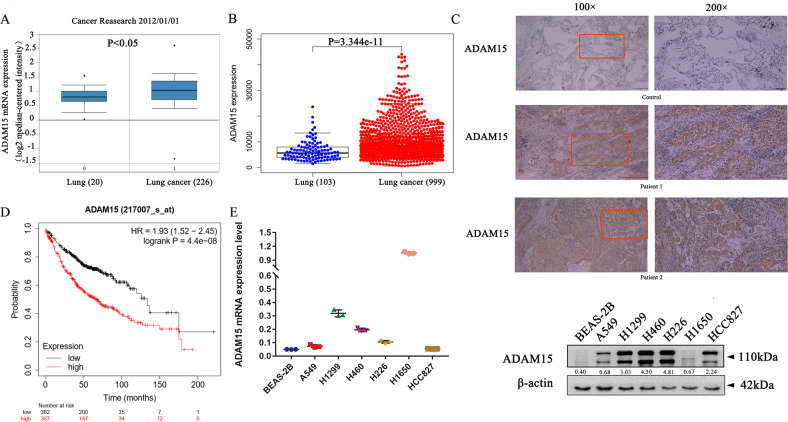
ADAM15 expression is upregulated in NSCLC cell lines. **A**, **B** Data obtained from the Oncomine database (http://www.oncomine.org) were analysed to compare the ADAM15 levels in 20 normal and 226 lung cancer tissues, and data obtained from TCGA database were analysed to compare the ADAM15 mRNA levels in 103 normal and 999 lung cancer tissues (https://portal.gdc.cancer.gov/). **C** NSCLC samples were immunostained with an anti-ADAM15 antibody. **D** Kaplan-Meier analysis of overall survival for ADAM15 expression in 719 adenocarcinoma samples. Kaplan–Meier plots were generated using Kaplan–Meier Plotter (http://www.kmplot.com). **E** qRT-PCR and western blot analyses of ADAM15 mRNA and protein levels in different non-small-cell lung cancer cells.

We used the Oncomine (http://www.oncomine.org) and TCGA (https://portal.gdc.cancer.gov/) databases to analyse ADAM15 expression, and the data showed that the mRNA expression of ADAM15 was higher in tumour tissues than in normal tissues (Fig. 1A, B). Immunohistochemistry (IHC) analysis was also carried out and showed the ADAM15 protein levels was higher in 8 paired NSCLC tissues than normal lung tissues (lung bullous tissue) (Figs. 1C, 6D and Fig. S4). Data extracted from Kaplan-Meier Plotter (http://www.kmplot.com) showed that high mRNA expression levels of ADAM15 were associated with poorer overall survival among 719 NSCLC patients (Fig. 1D). In addition, we also detected ADAM15 mRNA and protein expression levels in different NSCLC cell lines and the normal bronchial epithelial cell line BEAS-2B (Fig. 1E). We selected the A549 cell line (with lower ADAM15 expression) and H1299 cell line (with higher ADAM15 expression) for follow-up experiments.

### Overexpression or knockdown of ADAM15 affects NSCLC cell proliferation, migration and invasion

**Fig. 2:**
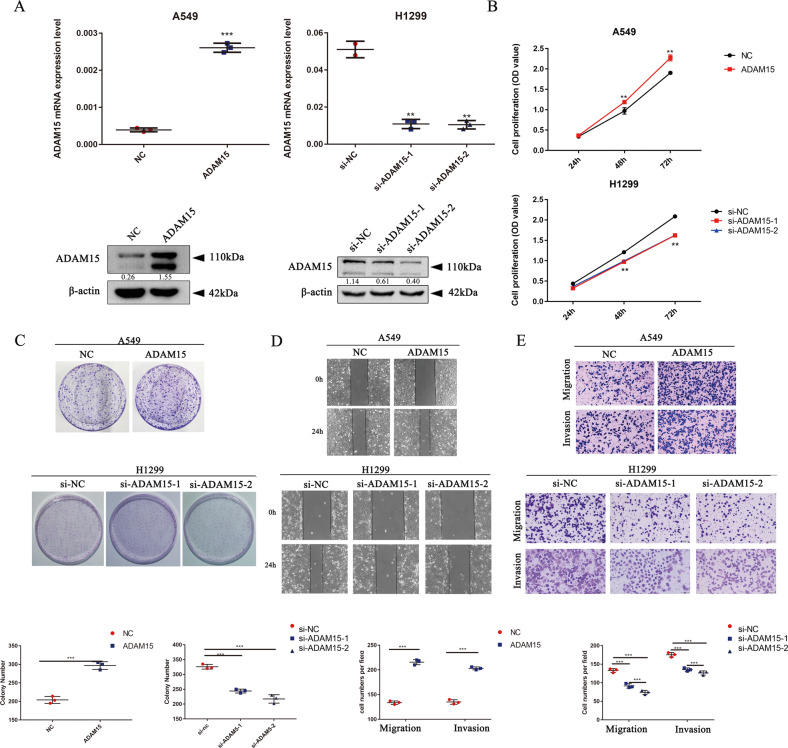
ADAM15 affects NSCLC cell proliferation, migration and invasion in ADAM15-overexpressing A549 cell lines and ADAM15-knockdown H1299 cell lines. **A** ADAM15 mRNA and protein levels were detected in ADAM15-overexpressing A549 cell lines and ADAM15-knockdown H1299 cell lines. **B**, **C** CCK-8 and clonogenic assays of cell proliferation in A549 and H1299 cell lines. **D** A wound healing assay was performed to evaluate the effect of ADAM15 in A549 and H1299 cells. **E** Images of the Transwell assay results for cell migration and invasion in A549 and H1299 cells. Bars represent the mean ± SEM from three independent experiments. *P < 0.05; **P < 0.01; ***P < 0.001.

To further investigate the function of ADAM15 in NSCLC cells, two NSCLC cell lines A549 (with lower ADAM15 expression) and H1299 (with higher ADAM15 expression), were chosen to construct stable ADAM15 overexpression and transient knockdown cell lines, respectively (Fig. 2A). Next, CCK-8 and clonogenic assays were performed to show that ADAM15 overexpression can promote cell proliferation, while ADAM15 knockdown can inhibit cell proliferation (Fig. 2B, C). The wound healing assay and the Transwell assay showed that ADAM15 can affect cell migration and invasion (Fig. 2D, E, Fig. S1). Additionally, the number of cells in S phase and the number of cells in G0/G1 phase changed, implying that ADAM15 may affect cell proliferation by regulating the cell cycle (Fig. S2).

### ADAM15 promotes NSCLC proliferation and migration and induces EGFR signalling pathway

**Fig. 3:**
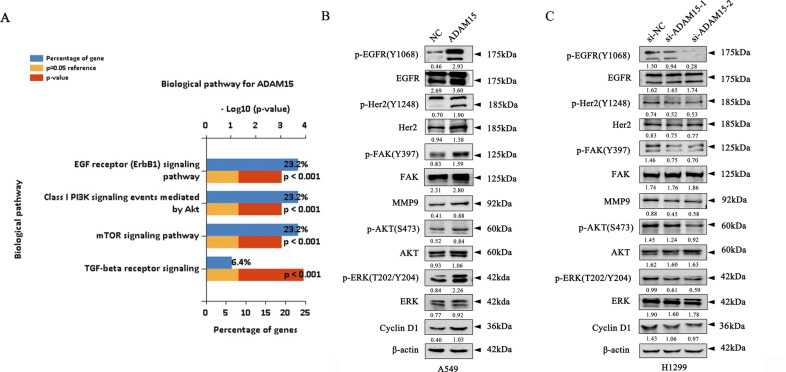
ADAM15 affects the EGFR-FAK signalling pathways in ADAM15-overexpressing A549 cell lines and ADAM15-knockdown H1299 cell lines. **A** Data from TCGA database and analysed by FunRich software (version 3.1.3). **B**, **C** Various protein mRNA and protein expression levels were measured by western blot analysis in ADAM15-overexpressing A549 cell lines and ADAM15-knockdown H1299 cell lines. β-actin was used as a loading control. *P < 0.05; **P < 0.01; ***P < 0.001.

To investigate the mechanism underlying ADAM15-mediated cell proliferation and metastasis, the data of lung adenocarcinoma gene expression were downloaded from TCGA database, and the genes with the co-expression coefficient of ADAM15 greater than 0.3 and *P* < 0.05 were screened by R studio software (version 3.1.3). Then the selected genes were input into Funrich software (version 3.1.3) for functional analysis. According to the LogFC value from high to low, we found that ADAM15 was mainly closely related to EGFR, PI3K and mTOR related signalling pathways (Fig. 3A), because the EGFR signalling pathways is a common upstream pathway and its abnormal activation is closely related to the occurrence and development of tumors, therefore, we examined the EGFR/Her2 pathways as candidate critical signalling pathways in our study. Western blot assays confirmed that the phosphorylation levels of EGFR and Her2 were upregulated in A549 cell lines when ADAM15 was overexpressed. Conversely, the phosphorylation levels of EGFR and Her2 were downregulated in H1299 cell lines after ADAM15 knockdown (Fig. 3B, C). Sequentially, we showed that the changes in downstream p-FAK, p-AKT, p-ERK, and cell cycle-associated cyclin D1 and MMP9 were due to ADAM15 expression manipulations, while the total FAK, AKT and ERK levels remained unchanged (Fig. 3B, C). To enhance the reliability of the above results, we repeated the above experiments in A549 cell lines and obtained similar results (Fig. S3A).

### Overexpression of ADAM15 in NSCLC cells promotes tumour growth via EGFR signalling

**Fig. 4:**
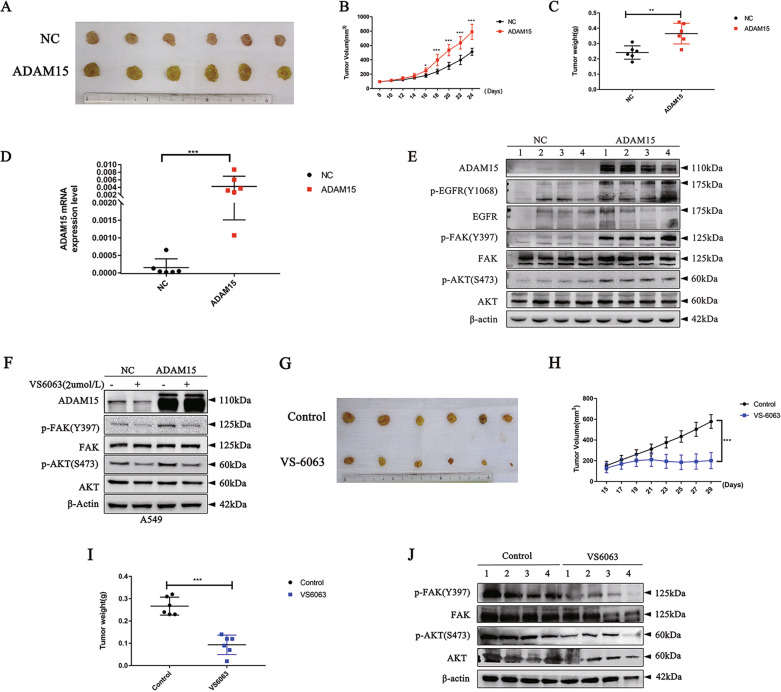
Effects of ADAM15 and FAK inhibitor defactinib (VS-6063) on NSCLC cell growth in vivo. **A** Representative images of tumours in these four groups. See the Methods section for details. **B** Tumour growth curves in mice on the indicated days. **C** Each tumour from indicated mice was weighed. **D**, **E** ADAM15 mRNA and protein expression in tumours was measured by qRT-PCR and western blot analyses. **F** Inhibition of FAK was carried out by using 2 μM defactinib (VS-6063) in A549 cell lines. **G** Representative images of tumours in these four groups. See the Methods section for details. **H** Tumour growth curves in mice on the indicated days. **I** Each tumour from the indicated mice was weighed. **J** Protein expression in tumours was measured by western blot analysis.

To further validate the effects of ADAM15 on tumour progression in vivo, control A549 cells and stable ADAM15-overexpressing A549 cells were inoculated into BALB/c athymic mice. Tumours formed from the cells with ADAM15 overexpression were much larger in size than those formed from the control cells (Fig. 4A). On the other hand, overexpression of ADAM15 promoted tumour growth, as evidenced by increased tumour volume and tumour weight (Fig. 4B, C). The tissues resected from the xenograft tumours were analysed to verify ADAM15 expression, and qRT-PCR showed that the ADAM15 mRNA levels in the ADAM15 overexpression group were increased compared with those in the control group (Fig. 4D). Western blot analysis also showed that the phosphorylation levels of EGFR and FAK were increased in the ADAM15 overexpression group (Fig. 4E). These results demonstrate a crucial role of EGFR signalling in ADAM15-mediated NSCLC cell proliferation and metastasis.

### Integrin signalling is involved in ADAM15-induced EGFR-FAK pathway activation

**Fig. 5:**
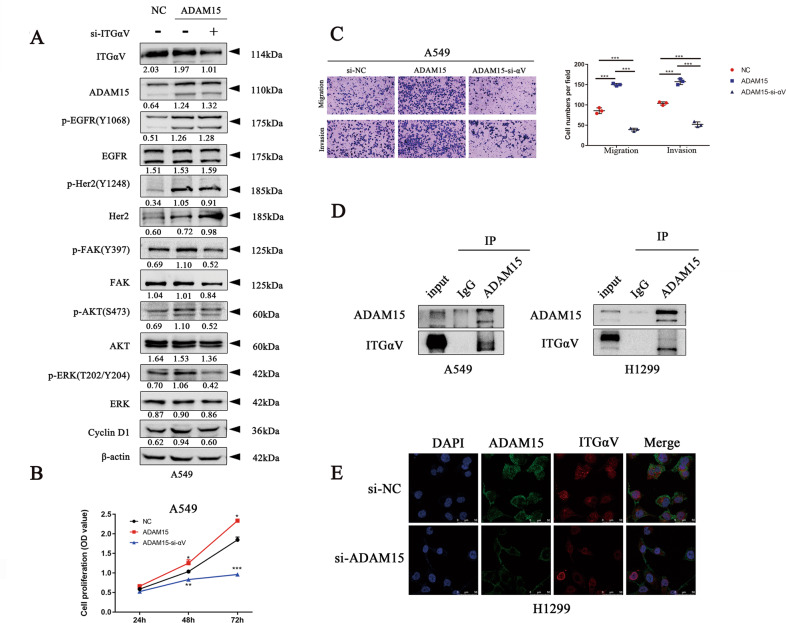
ADAM15 affects the FAK signalling pathway through integrin αV. **A** Control and ADAM15-overexpressing stable A549 cells were treated with the indicated siRNA (si-αV compared with si-NC) followed by western blotting. **B**, **C** Control and ADAM15-overexpressing stable A549 cells were treated with the indicated siRNA (si-αV compared with si-NC), followed by CCK8 and Transwell assays. **D** Co-immunoprecipitation of ADAM15 and integrin αV is shown. Protein was extracted from parental A549 and H1299 cells using a specific monoclonal antibody. **E** Immunofluorescence staining of ADAM15 and integrin αV co-expression in ADAM15-knockdown cells compared to control cells (Scale bar: 50 μm). *P < 0.05; **P < 0.01; ***P < 0.001.

Gene expression data in lung cancer cell lines were downloaded from the CClE database (https://portals.broadinstitute.org/ccle) and analysed using GSEA software. We found that ADAM15 is closely related to cell adhesion (Fig. S3B). The disintegrin domain of ADAM15 contains an RGD binding site, which enables interaction with αVβ3 integrin. A previous study reported the co-expression of ADAM15 and αVβ3 in lung adenocarcinoma [19], therefore, we examined whether integrin signalling is involved in the ADAM15-induced EGFR signalling pathway. To investigate the mechanism, we knocked down integrin αV expression in ADAM15-overexpressing cell lines. We found that αV knockdown reduced the increase in the phosphorylation levels of FAK, AKT and ERK caused by ADAM15 overexpression; however, it did not affect the phosphorylation levels of EGFR/Her2 (Fig. 5A). Indeed, many studies have reported that αV directly affects the FAK signalling pathway [20–23]. Then, we found that knockdown of integrin αV inhibited cell proliferation, migration and invasion and that ADAM15 overexpression rescued the decreased abilities (Fig. 5B, C). Next, we examined complexes of ADAM15 and αV using coimmunoprecipitation (Fig. 5D). Immunofluorescence experiments also showed that ADAM15 and integrin αV were co-expressed (Fig. 5E). Therefore, we believe that ADAM15 can affect the FAK signalling pathway by affecting the expression of integrin αV, however, it is worth considering how ADAM15 affected the EGFR signalling pathway.

### ADAM15 and CD151 are co-expressed in NSCLC

**Fig. 6:**
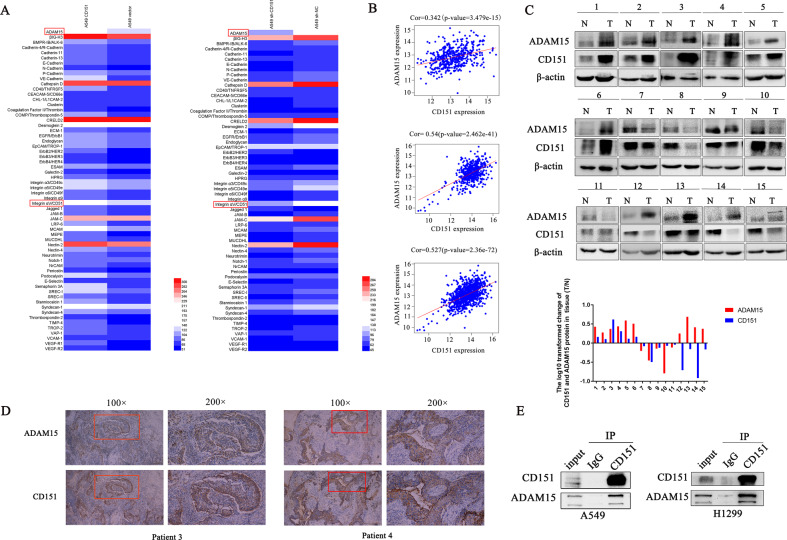
ADAM15 and CD151 co-expression in NSCLC. **A** Hierarchical cluster analysis of microarray data (heat map) was performed to show differentially expressed proteins of A549 cells in different groups. Red and blue indicate high and low protein expression levels, respectively. **B** Data obtained from TCGA database (https://portal.gdc.cancer.gov/) were analysed to detect the correlation between ADAM15 and CD151 mRNA levels in lung adenocarcinoma, lung squamous cell carcinoma and total lung cancer cell lines. **C** Western blot analysis of the ADAM15 and CD151 protein levels in 15 randomly selected NSCLC tissues and paired noncancerous lung tissues. **D** NSCLC samples were co-immunostained with an anti-CD151 antibody and anti-ADAM15 antibody. **E** Co-immunoprecipitation of ADAM15 and CD151 is shown. Protein was immunoprecipitated and detected from parental A549 and H1299 cells using a specific monoclonal antibody. Bars represent the mean ± SEM from three independent experiments. *P < 0.05; **P < 0.01; ***P < 0.001.

Based on our team’s research on CD151 [14], a Proteome Profiler Array of CD151 knockdown and overexpression cell lines was carried out to compare the density of relative proteins. And the heat map showed that ADAM15 exhibited the largest fold changes among the selected genes due to CD151 expression manipulation (Fig. 6A). Therefore, we examined the correlation between ADAM15 and CD151 in our study. Data extracted from TCGA database showed that ADAM15 mRNA was positively correlated with the CD151 mRNA levels in NSCLC (Fig. 6B).

To verify the relationship between ADAM15 and CD151 in NSCLC tissue samples, we valuated 15 paired NSCLC tissues and adjacent noncancerous lung tissues (Fig. 6C). The clinicopathological parameters of these 15 paired samples are shown in Table S1 (one case of NSCLC was excluded because it originated from intestinal metastasis). Interestingly, we found that the CD151 protein level (T/N) and ADAM15 protein level (T/N) were positively correlated in 11 pairs of the samples, accounting for 73.3% of 15 pairs of tissues (Fig. 6C). Then, immunohistochemistry (IHC) analysis was also carried out to evaluate the ADAM15 and CD151 protein levels in paired NSCLC tissues (Fig. 6D, Fig. S4), we found that ADAM15 and CD151 were co-expressed. The clinicopathological parameters of paired NSCLC samples are shown in Table S2. Finally, we identified that ADAM15 and CD151 have a certain correlation using coimmunoprecipitation in the A549 and H1299 cell lines (Fig. 6E).

### ADAM15 cooperate with CD151 to affect the integrin α3/α6-EGFR-FAK signalling pathway

**Fig. 7:**
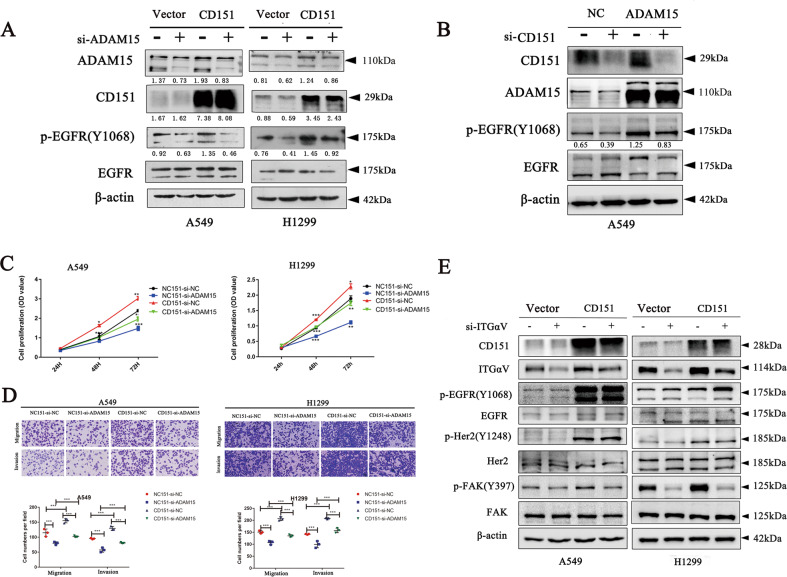
ADAM15 and CD151 cooperate to affect the integrin-related EGFR signalling pathway. **A** Control and CD151-overexpressing stable A549 and H1299 cells were transfected with the indicated siRNA (si-ADAM15 compared with si-NC) followed by western blotting. β-actin was used as a loading control. **B** Control and ADAM15-overexpressing stable A549 cells were transfected with the indicated siRNA (si-CD151 compared with si-NC) followed by western blotting. β-actin was used as a loading control. **C**, **D** CD151-overexpressing stable A549 and H1299 cells were transfected with the indicated siRNA (si-ADAM15 compared with si-NC) followed by CCK-8 and Transwell assays. **E** Control and CD151-overexpressing stable A549 and H1299 cells were treated with the indicated siRNA (si-αV compared with si-NC) followed by western blotting. β-actin was used as a loading control. *P < 0.05; **P < 0.01; ***P < 0.001.

To clarify the correlation between ADAM15 and CD151, we found that transfection with ADAM15 siRNA reduced the increase in the phosphorylation levels of EGFR caused by CD151 overexpression (Fig. 7A). We next interfered with CD151 in ADAM15-overexpressing cells and found that transfection with CD151 siRNA reduced the increase in the phosphorylation levels of EGFR caused by ADAM15 overexpression (Fig. 7B). Then we inhibited ADAM15 in CD151-overexpressing cells and found that CD151 overexpression rescued the decreased cell proliferation migration and invasion inhibited by ADAM15 knockdown (Fig. 7C, D). Indeed, our previous study has demonstrated CD151 regulates the EGFR-FAK signalling pathway by affecting integrin in NSCLC [14] and Pengcheng Zhou et al. also have proven that CD151 regulates the EGFR signalling pathway by affecting integrin in glioblastoma [24]. Thus, we speculate that ADAM15 and CD151 cooperate to regulate integrin-related EGFR signalling pathways.

**Fig. 8:**
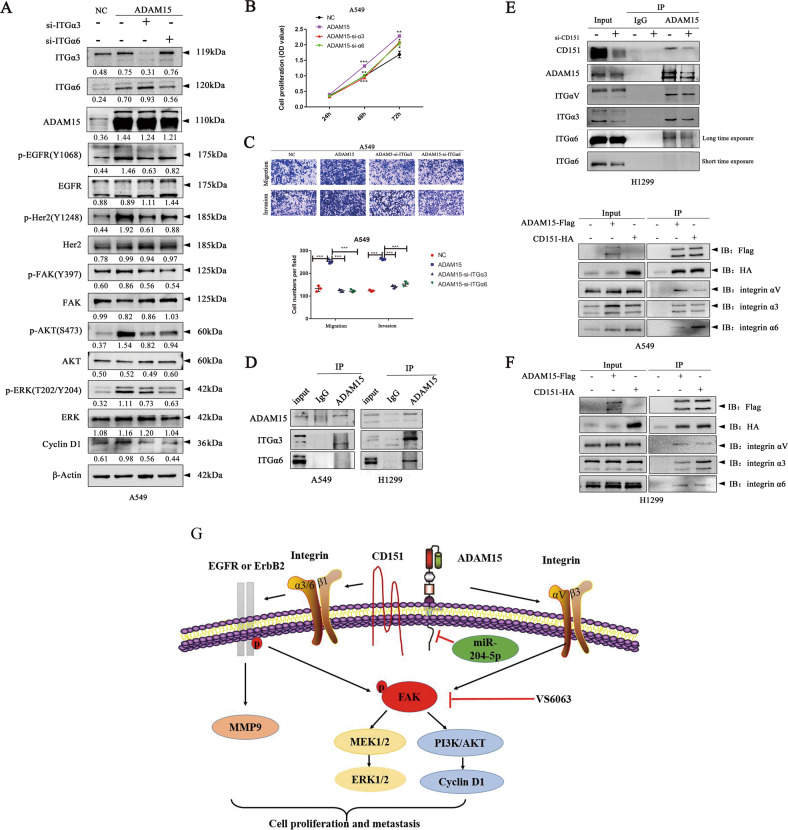
ADAM15 affects the EGFR signalling pathway through integrin α3/α6. **A** Control and ADAM15-overexpressing stable A549 cells were treated with the indicated siRNA (si-α3/α6 compared with si-NC) followed by western blotting. β-actin was used as a loading control. **B**, **C** Control and ADAM15-overexpressing stable A549 cells were treated with the indicated siRNA (si-α3//α6 compared with si-NC), followed by CCK8 and Transwell assays. **D** Co-immunoprecipitation of ADAM15 and integrin α3/α6 is shown. Protein was immunoprecipitated and detected from parental A549 and H1299 cells using a specific monoclonal antibody. **E** Co-immunoprecipitation of ADAM15 and integrins were shown, protein were immunoprecipitation from lysates of control and CD151 knockdown H1299 cells using a specific monoclonal antibody. **F** The Flag-tagged ADAM15 vector and HA-tagged CD151 vector were cotransfected into A549 and H1299 cells for 48 h. The protein were immunoprecipitated using agarose beads with anti-Flag antibody. *P < 0.05; **P < 0.01; ***P < 0.001. **G** Schematic illustration of the functional roles of ADAM15, CD151 and integrin complexes in NSCLC.

Since integrin α3/α6 has been reported to form a complex with CD151 [25–27] and ADAM15 was also co-expressed with CD151, we therefore explored whether α3/α6 is involved in the EGFR signalling pathway induced by ADAM15. We first confirmed that integrin αV does not affect the EGFR/Her2 signalling pathway but directly affects the FAK signalling pathway (Fig. 7E). To further investigate the mechanism, we next found that α3/6 knockdown reduced the increase in the phosphorylation levels of EGFR/Her2, FAK and downstream proteins caused by ADAM15 overexpression (Fig. 8A). Furthermore, knockdown of integrin α3/α6 attenuated the enhancement of cell proliferation, migration and invasion abilities caused by ADAM15 overexpression (Fig. 8B, C). Then, we performed co-immunoprecipitation to show that ADAM15 can interact with α3/α6 (Fig. 8D), furthermore, we found CD151 interference in NSCLC indeed decreased the combination of ADAM15 and integrin α3/α6, but the combination of ADAM15 and integrin αV was not reduced (Fig. 8E), the results proved ADAM15 regulate integrin α3/α6 by cooperating with CD151, but regulate integrin αV is not affected by CD151. Furthermore, co-immunoprecipitation experiment confirmed a specific interaction of ADAM15 with integrin αV, and CD151 with integrin α3/α6 (Fig. 8F). Therefore, we believe that ADAM15 and CD151 cooperate to affect the EGFR signalling pathway by binding to integrin α3/α6.

### FAK inhibitor defactinib (VS-6063) can suppress tumour growth induced by overexpression of ADAM15

Based on the above findings, we believe that FAK is the core molecule of the two ADAM15 signalling pathways that regulate downstream proteins through integrins. Therefore, we first verified the effect of the FAK inhibitor defactinib (VS-6063) on proliferation of NSCLC (Fig. 4F). Then, tumours formed from the ADAM15-overexpressing cells treated with defactinib (VS-6063) were much smaller in size than those formed from the control cells (Fig. 4G). Tumour growth was slower in the defactinib (VS-6063) treatment group than in the control group, as evidenced by the tumour volume and tumour weight curve (Fig. 4H, I). Western blot also showed that defactinib (VS-6063) resulted in a large decrease in the p-FAK and p-AKT levels in the defactinib (VS-6063) treatment group compared with in the control group (Fig. 4J). Taken together, the above findings suggest the potential of FAK inhibitor therapy to improve the clinical outcome of NSCLC patients.

### MiR-204-5p directly targets ADAM15 and regulates the proliferation of NSCLC

Because the mechanism underlying the high expression of ADAM15 in NSCLC is unclear and miRNAs directly regulate gene expression, we aimed to explore whether the increase in ADAM15 in NSCLC was caused by miRNAs. We used multiple databases to predict miRNAs and screen out 5 most possible miRNAs, among which miR-204-5p was confirmed negatively related to ADAM15 (Fig. S5A, B). The TargetScan Human (http://www.targetscan.org/) database was then used to predict the possible binding sites for miR-204-5p in the 3′-UTR of ADAM15 mRNA (Fig. S5C). Indeed, miR-204-5p plays a vital role in NSCLC and has been reported to mediate the apoptosis of NSCLC cells [28]. Data extracted from the TCGA (https://portal.gdc.cancer.gov/) database showed that the expression of miR-204-5p was lower in tumour tissues (Fig. S5D). Then, we identified that the miR-204-5p mRNA levels were lower in NSCLC cell lines (Fig S5E). Next, we used Kaplan-Meier Plotter (http://www.kmplot.com) to showed that low miR-204-5p level was associated with poorer overall survival (Fig. S5F). A dual-luciferase reporter vector containing the ADAM15 3′-UTR seed region specific to miR-204-5p or the corresponding mutant sequence was used to confirm the direct binding between miR-204-5p and ADAM15 (Fig. S5E). Furthermore, the RNA and protein levels of ADAM15 were negatively regulated by miR-204-5p (Fig. S5G–J), and other candidate miRNAs were ruled out (Fig. S5L, N). The effects of miR-204-5p on NSCLC cell proliferation were also investigated by CCK-8 and clonogenic assays (Fig. S5K, M). The above findings show that miR-204-5p affects cell proliferation by targeting ADAM15.

## Discussion

In our study, we showed that high ADAM15 expression was associated with poor prognosis of NSCLC, which suggesting the indispensable role of ADAM15 in NSCLC progression. Furthermore, CD151 is a widely recognized oncogene in a variety of cancers. Interestingly, we found that ADAM15 and CD151 were co-expressed, and the presence of ADAM15 affected the integrin-related EGFR signalling pathway by cooperating with CD151. Together, we demonstrated that ADAM15 affects the FAK and EGFR signalling pathways by binding to integrin αV and integrin α3/α6 respectively. A summary diagram of the pathways in this study was showed in Fig. 8G.

It has been reported that ADAM15 is upregulated in many malignant tumours, and the high expression of ADAM15 is associated with the progression of tumours [29]. However, in some cancers, such as colon cancer, the expression of ADAM15 inhibits cancer metastasis and is associated with poor prognosis in patients with colon cancer [30]; In our study, we found that ADAM15 was an independent prognostic factor and that the high expression of ADAM15 correlated with the overall survival (OS) of lung cancer patients. Therefore, we hypothesized that ADAM15 plays a dual role in the context of various types of cancer.

The interaction of CD151 and EGFR and Her2 has been reported [14, 31], and along with the correlation between ADAM15 and CD151, we found that ADAM15 cooperates with CD151 to regulate the EGFR signalling pathway. FAK is a nonreceptor intracellular tyrosine kinase that plays important roles in the aspects of cell adhesion, proliferation, and migration in tumours [32]. It can be activated by transmembrane integrins and many other growth factors, such as EGFR, linking to the formation and turnover of focal adhesions, which then phosphorylates and activates downstream signalling pathways [33]. Consistently, in this study, we confirmed that ADAM15 could affect the progression of NSCLC by regulating the EGFR-FAK-AKT/ERK signalling pathways. The matrix metalloproteinase (MMP) family members can degrade various types of collagen and gelatine, which infers that they are closely related to the migration and invasion of tumours [34, 35]. In our study, we also demonstrated that ADAM15 regulated the migration and invasion of tumours by affecting MMP9.

Integrins are key regulators between cells and the microenvironment which play a vital role in cancer proliferation and metastasis [36]. Integrin αV is a large family of RGD-binding integrins. In addition to being involved in cell proliferation, migration, differentiation, apoptosis and adhesion [36], RGD-binding integrins have been identified as attractive in vivo targets for the molecular imaging of tumours [37]. Recently, we demonstrated that integrin αV is a key part of the drug resistance mechanism of EGFR-mutated non-small-cell lung cancer [38]. ADAM15 contains a consensus RGD site in its disintegrin domain, which can play a role in cell adhesion by binding to integrin αVβ3 [7, 8, 30], and the colocalization of ADAM15 and αVβ3 integrin has been demonstrated in lung carcinomas [19]. CD151 has been reported to be related to integrin α3/α6 [39, 40]. Integrin α3/α6 is a large family of laminin-binding integrins and has also been reported to be involved in the occurrence and progression of a variety of cancers [41]. In this study, we firstly proved the co-expressed of ADAM15 and CD151, based on the above, we explored whether ADAM15 affects the EGFR signalling pathway through integrin αV/α3/α6. In present study, we found that the regulation of integrin by ADAM15 is divided into two pathways: the ADAM15-αV-FAK pathway and the ADAM15-α3/α6-EGFR-FAK pathway. FAK is the core molecule, and the FAK signalling pathway is common downstream of the two integrin signalling pathways as our study suggested. We used the FAK inhibitor defactinib (VS-6063) in vivo and in vitro, and the FAK signalling pathway of tissues and cells was significantly inhibited. Indeed, FAK, a very important cancer-promoting molecule in cancer, is involved in a variety of cancer signalling pathways [42, 43], and αV-FAK has been demonstrated to be related to the resistance of EGFR mutated non-small-cell lung cancer [38].

In recent years, increasing studies have shown that microRNAs were closely related to the occurrence and development of tumours, and microRNA can regulate the expression of target genes by binding to the mRNA of target genes [44]. MiR-204-5p has been proven to have an anticancer effect in a variety of cancers such as gastric cancer, breast cancer [45, 46]. The dual-luciferase reporter assay confirmed that miR-204-5p has binding sites with ADAM15, and miR-204-5p can inhibit ADAM15-induced proliferation of NSCLC, which further confirms the tumour promoting function of ADAM15 and provides more clues to the regulation network of ADAM15.

## Conclusions

In conclusion, our study proved that high expression of ADAM15 correlates with a poor outcome for NSCLC patients, and shed light on the mechanistic interaction between ADAM15 and CD151 in NSCLC carcinogenesis. This FAK inhibitor defactinib (VS-6063) provided a new insight into therapy strategies for patient of NSCLC with high ADAM15 expression.

## Supplementary information

Fig S1

Fig S2

Fig S3

Fig S4

Fig S5

Table S1

Table S2

Supplementary materials.

Original Data File

## Data Availability

The datasets used and/or analysed during the current study are available from the corresponding author on reasonable request.
