# Supplementary figures and images for: Integrin α3/α6 and αV are implicated in ADAM15-activated FAK and EGFR signalling pathway individually and promote non-small-cell lung cancer progression
Source: Cell Death Dis. 2022 May 21;13(5):486. doi: 10.1038/s41419-022-04928-0 (PMC9124216; doi:10.1038/s41419-022-04928-0)

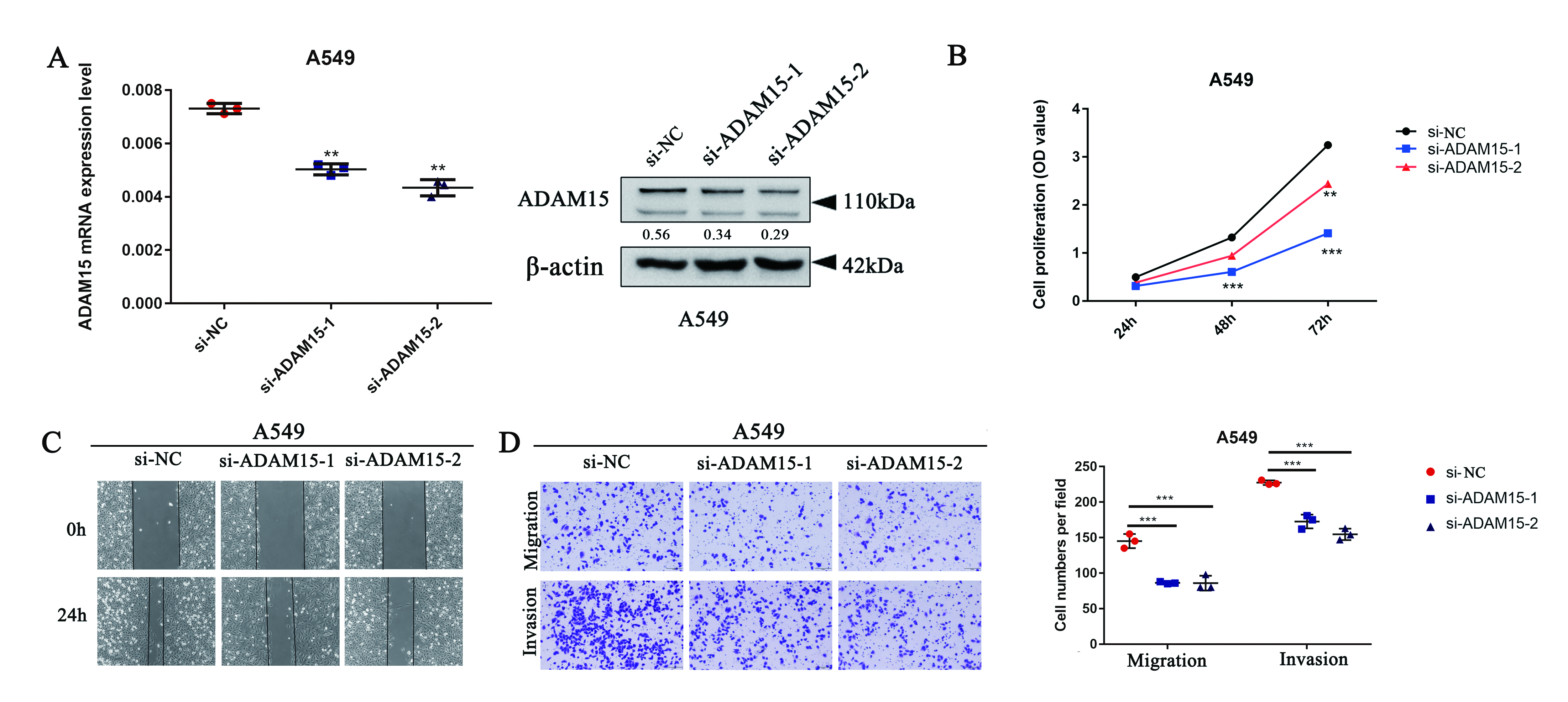

Supplement: Supplementary file 1 — Fig S1 [file 41419_2022_4928_MOESM1_ESM.tif]

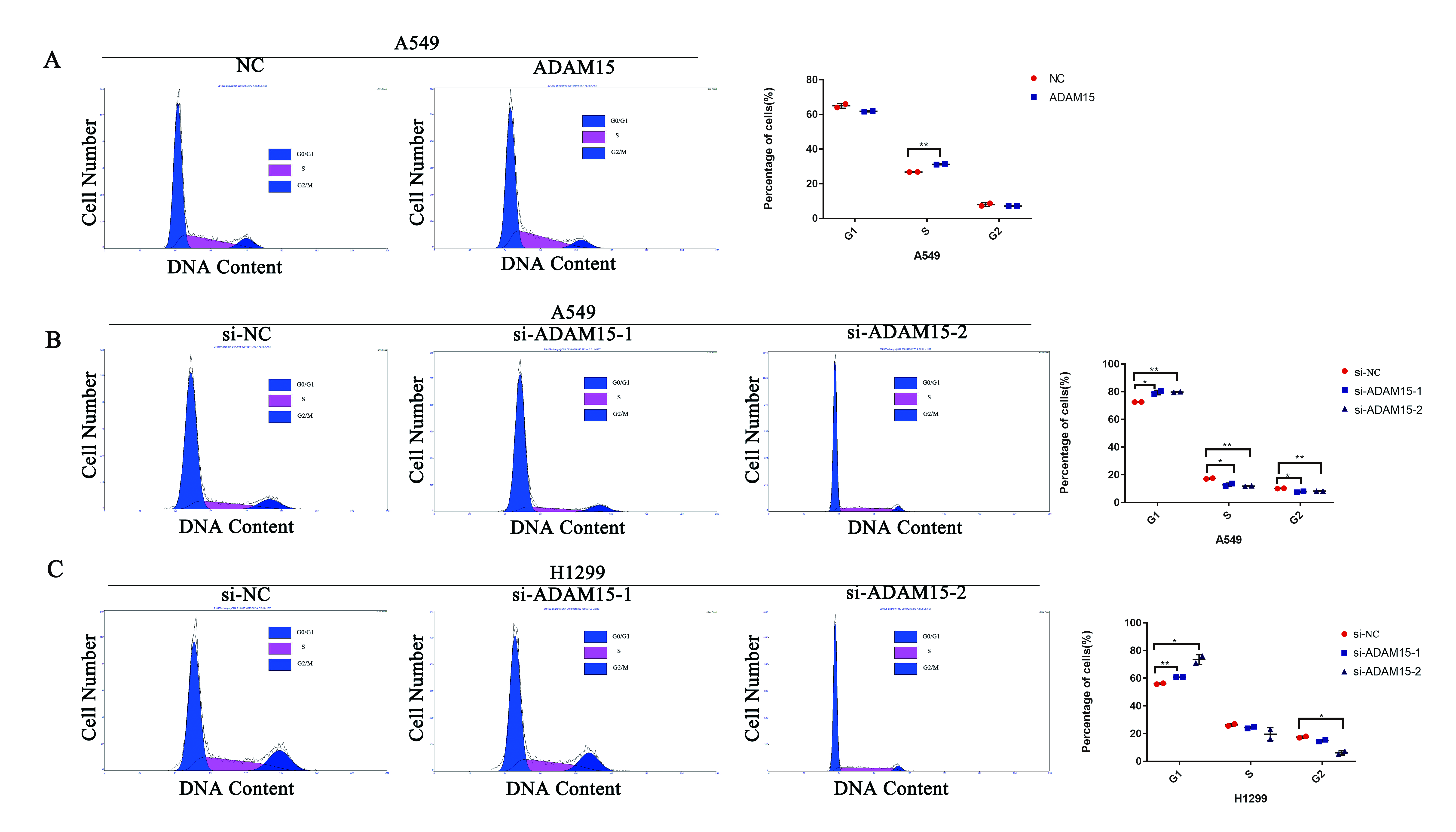

Supplement: Supplementary file 2 — Fig S2 [file 41419_2022_4928_MOESM2_ESM.tif]

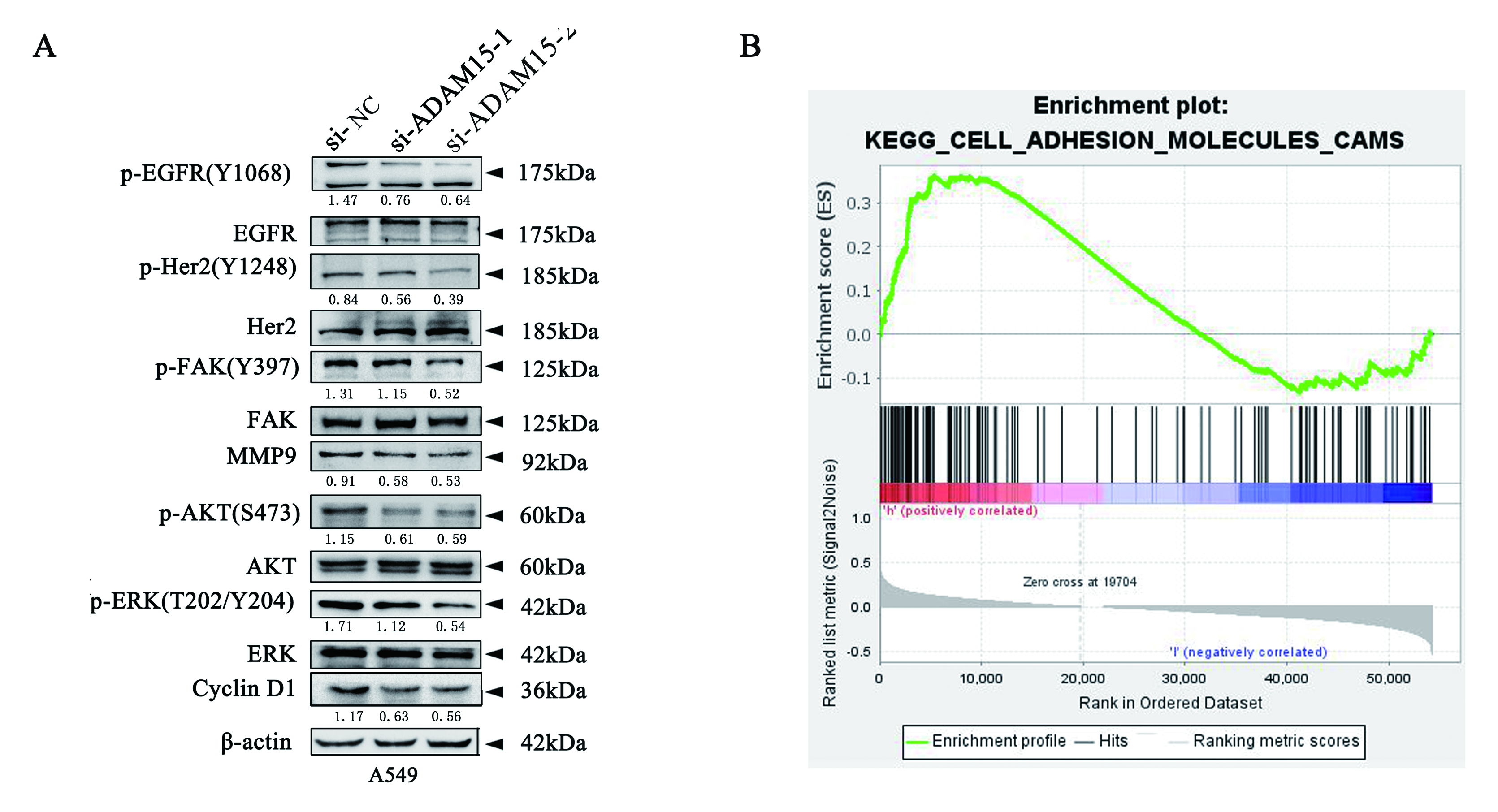

Supplement: Supplementary file 3 — Fig S3 [file 41419_2022_4928_MOESM3_ESM.tif]

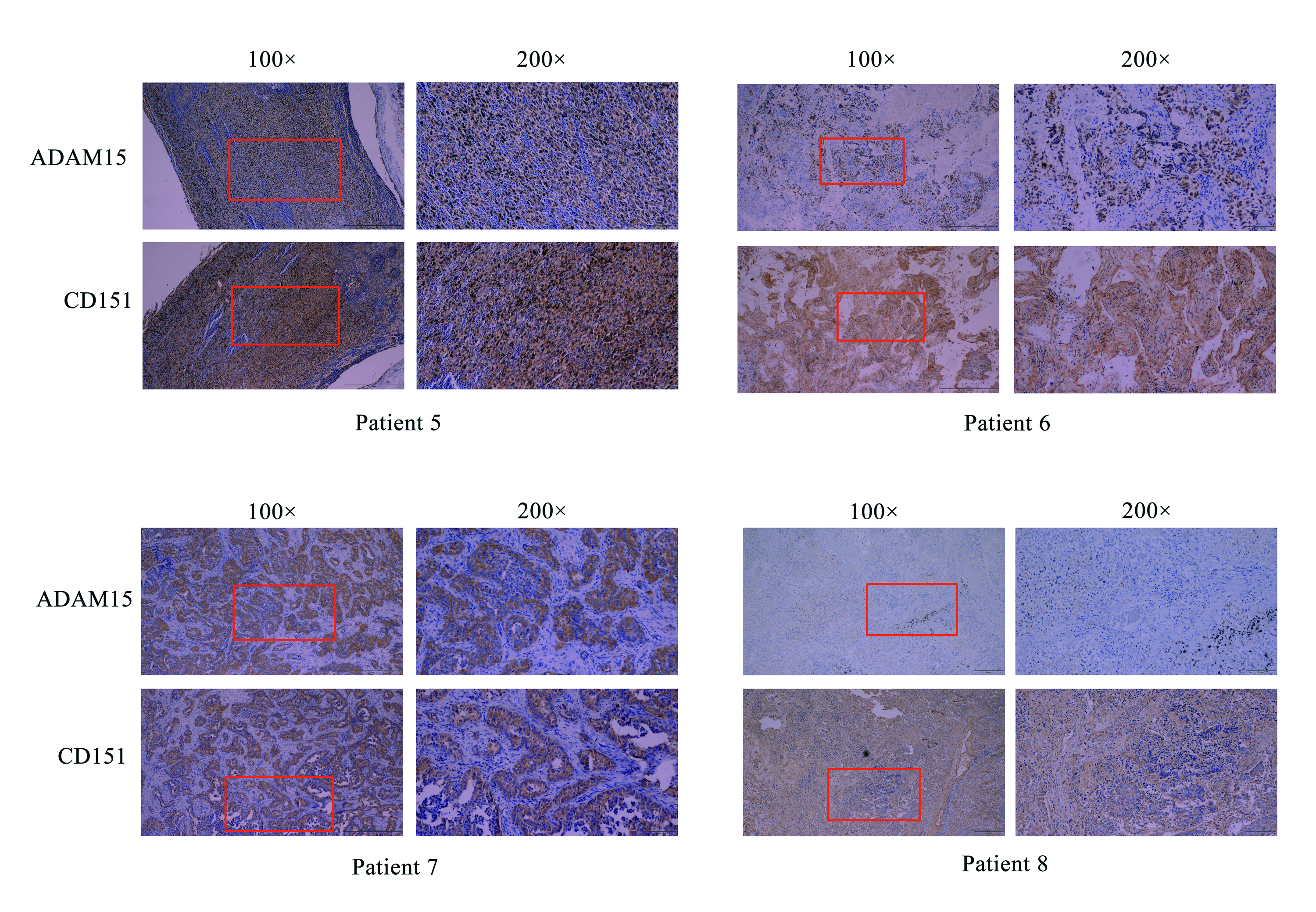

Supplement: Supplementary file 4 — Fig S4 [file 41419_2022_4928_MOESM4_ESM.tif]

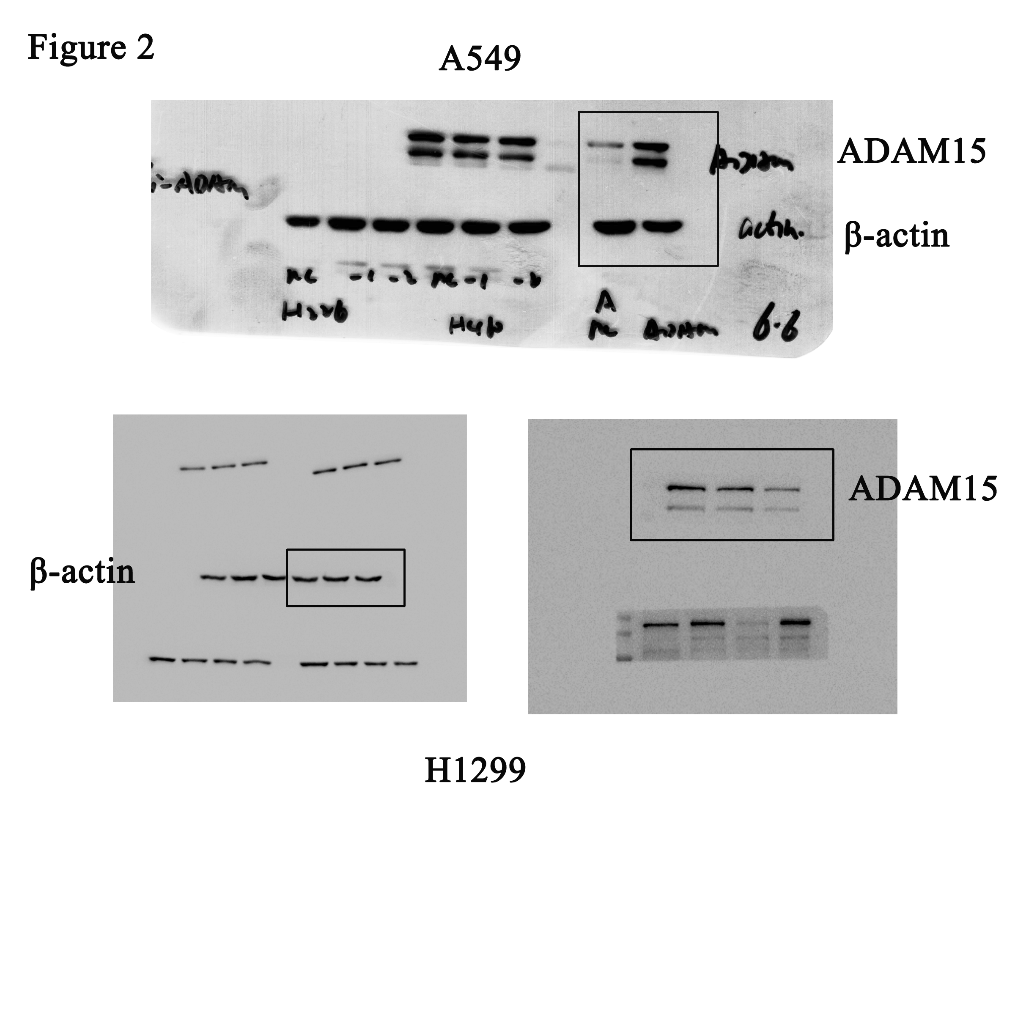


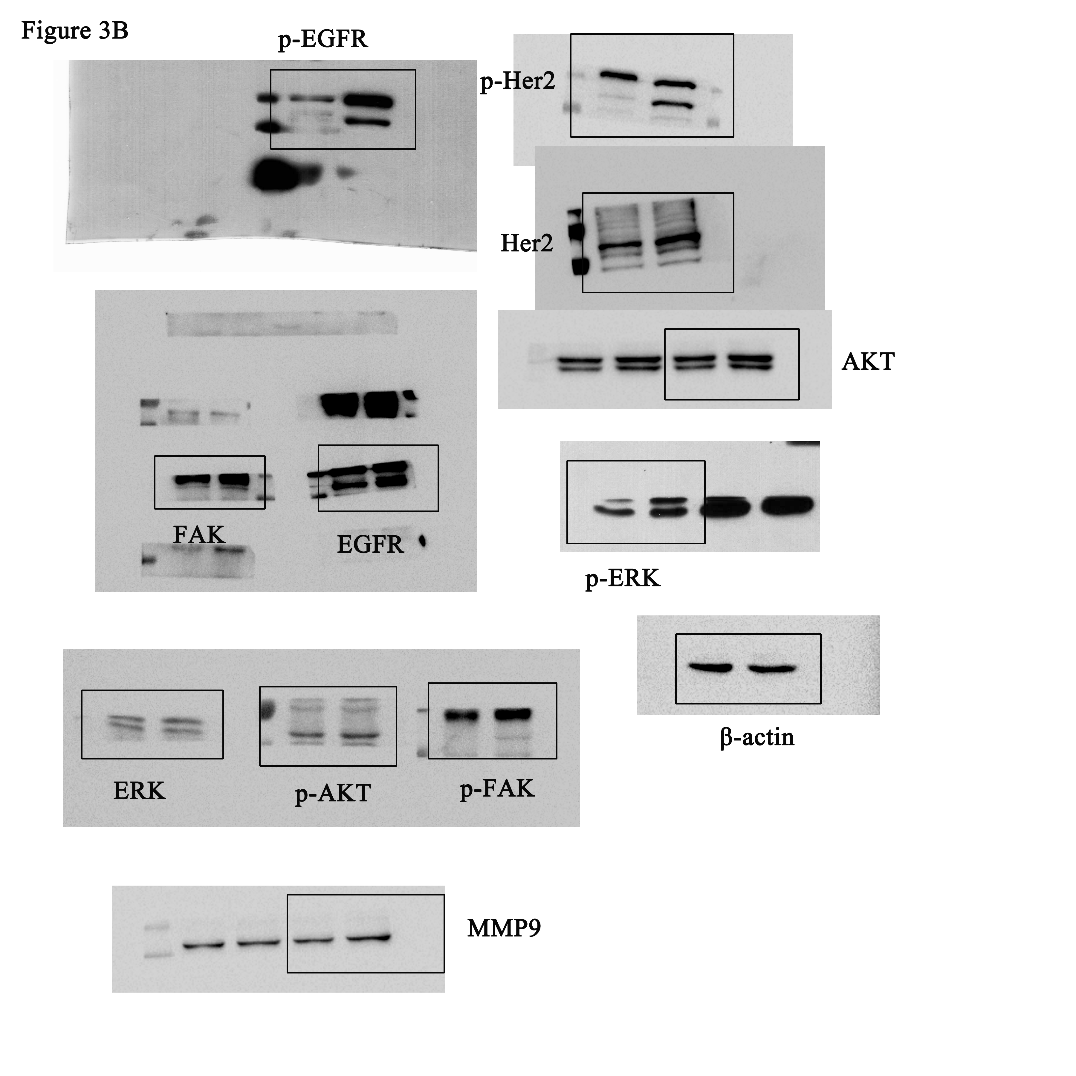


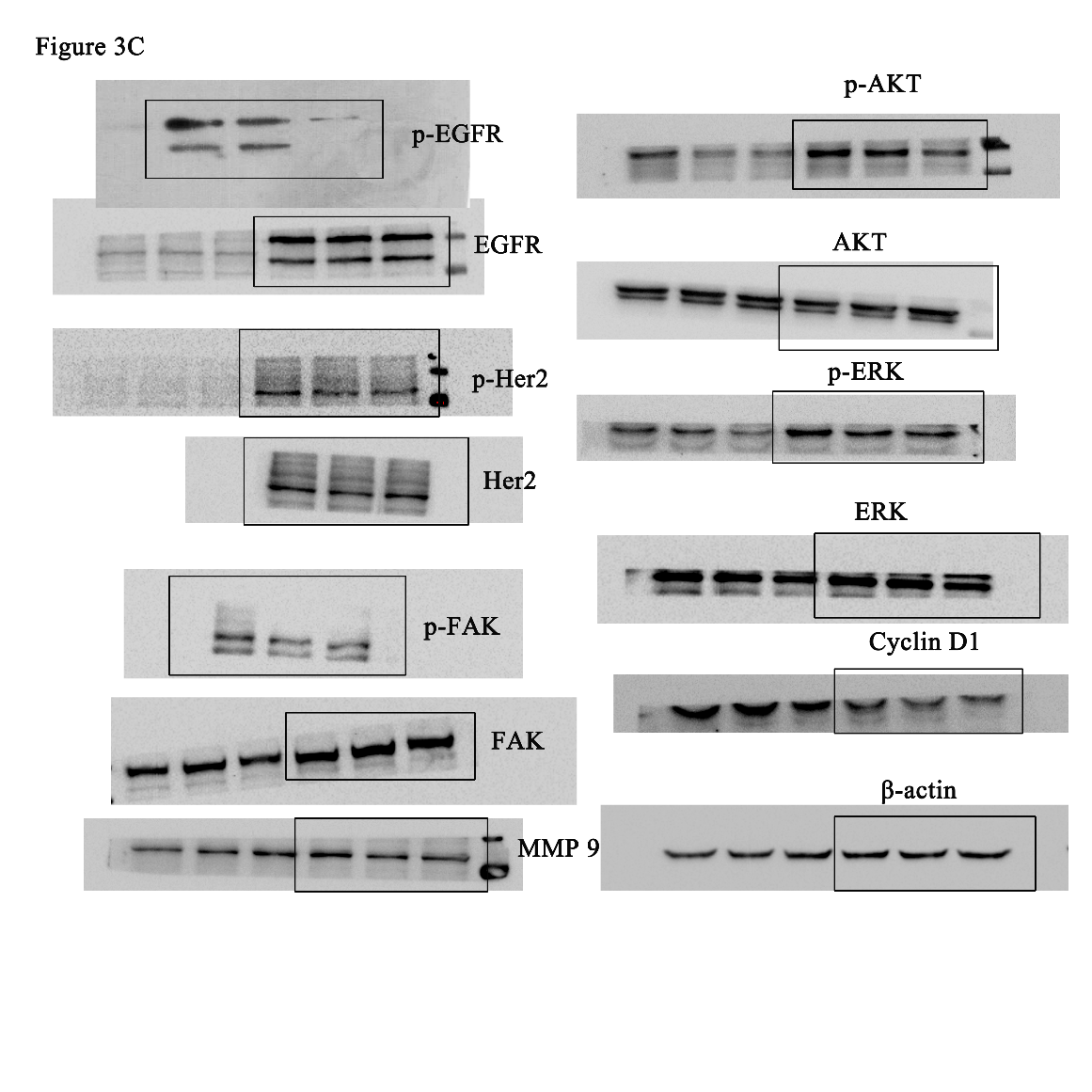


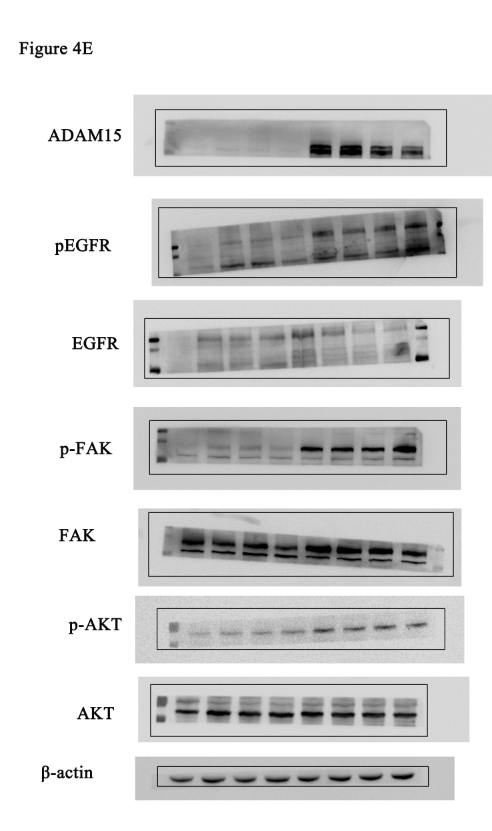


**Figure 4F**


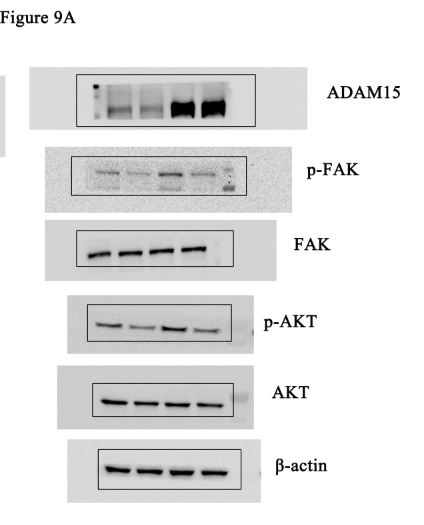


**Figure 4J**


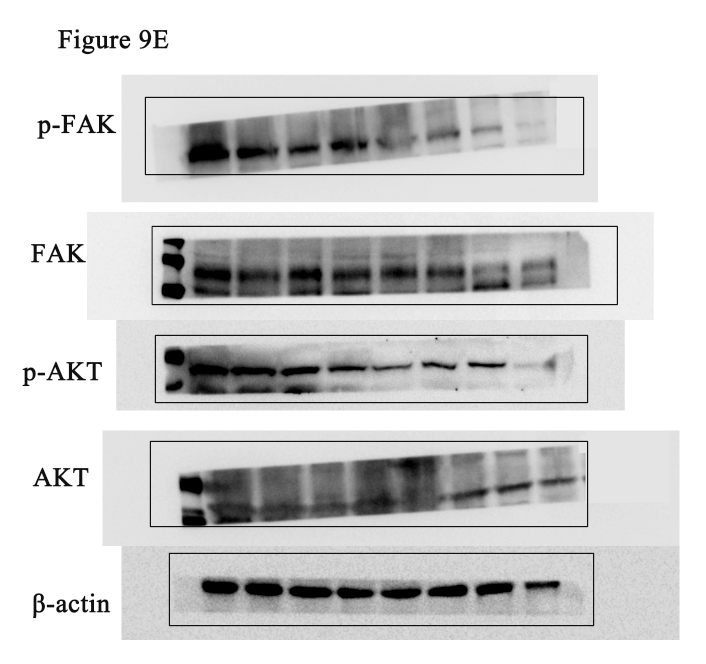


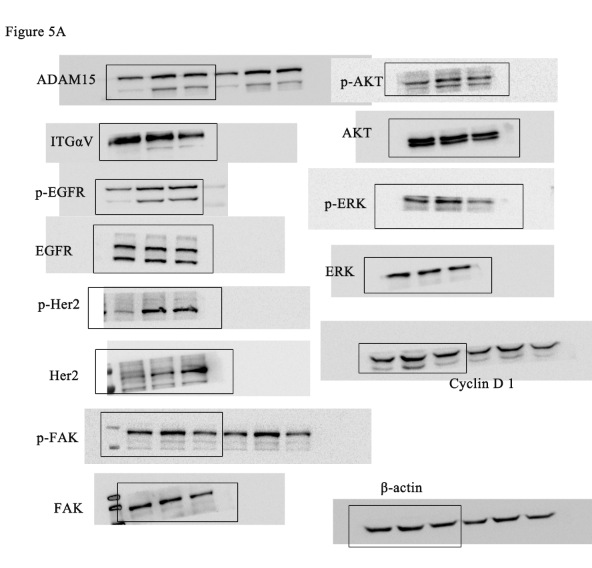


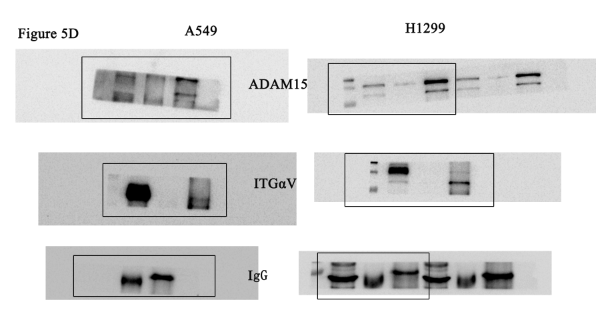


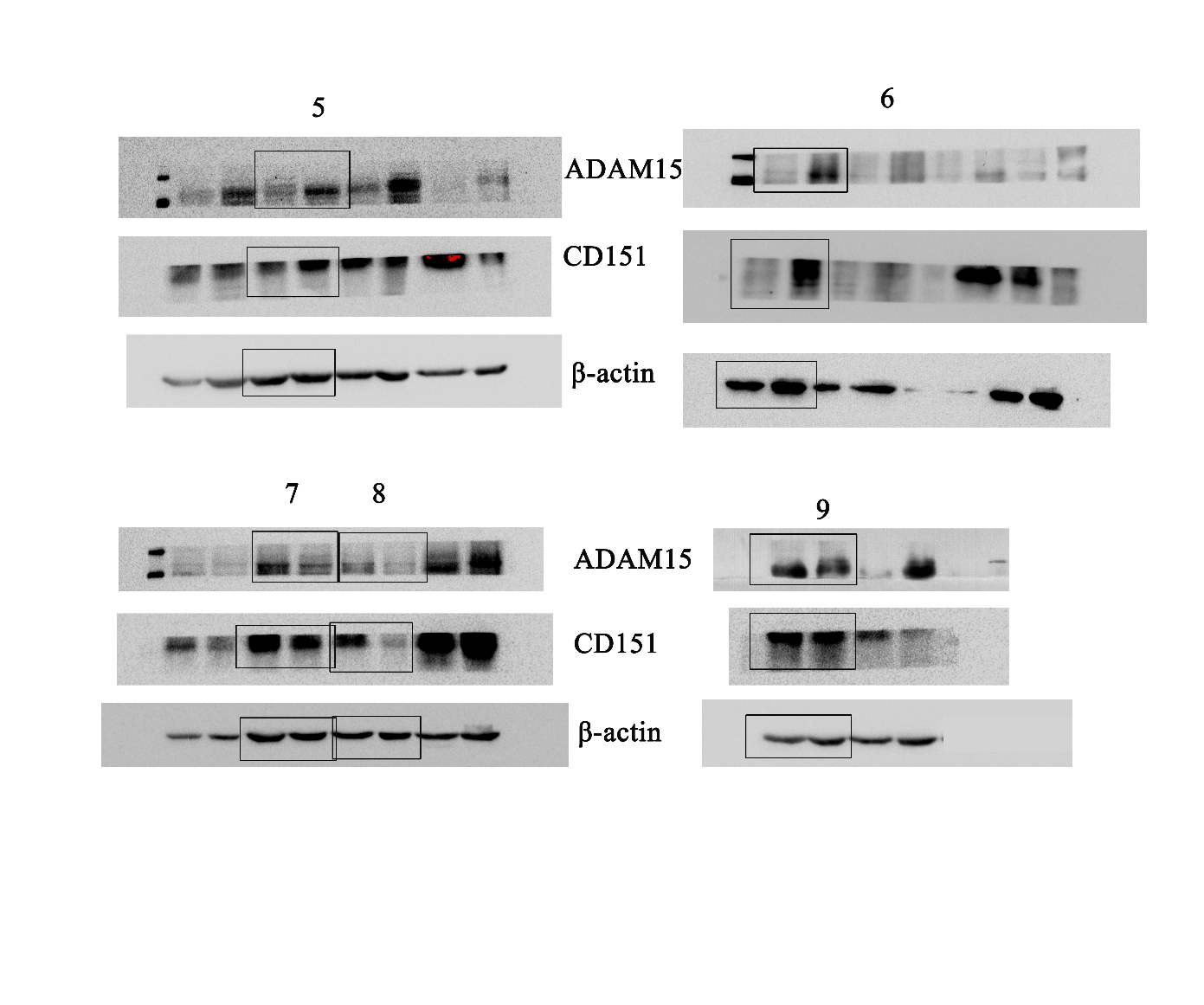

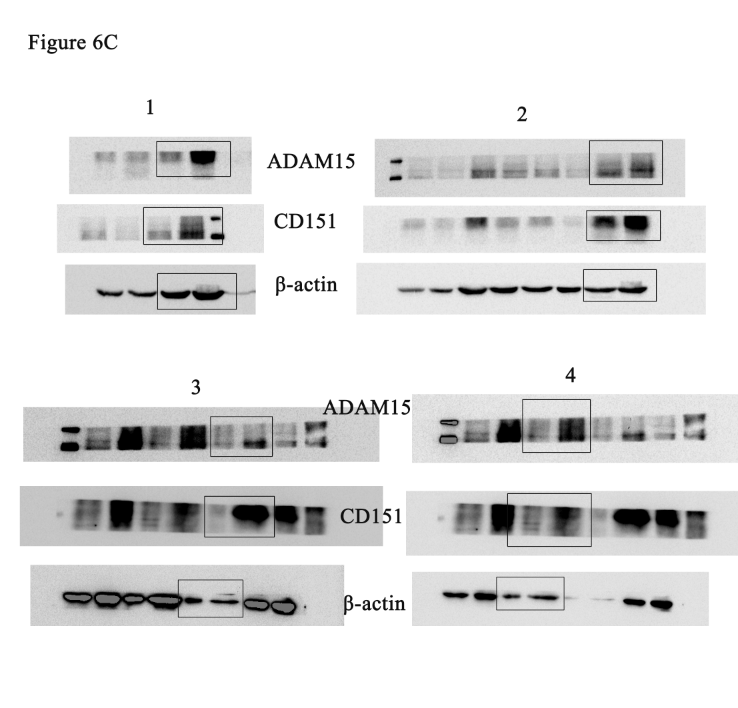


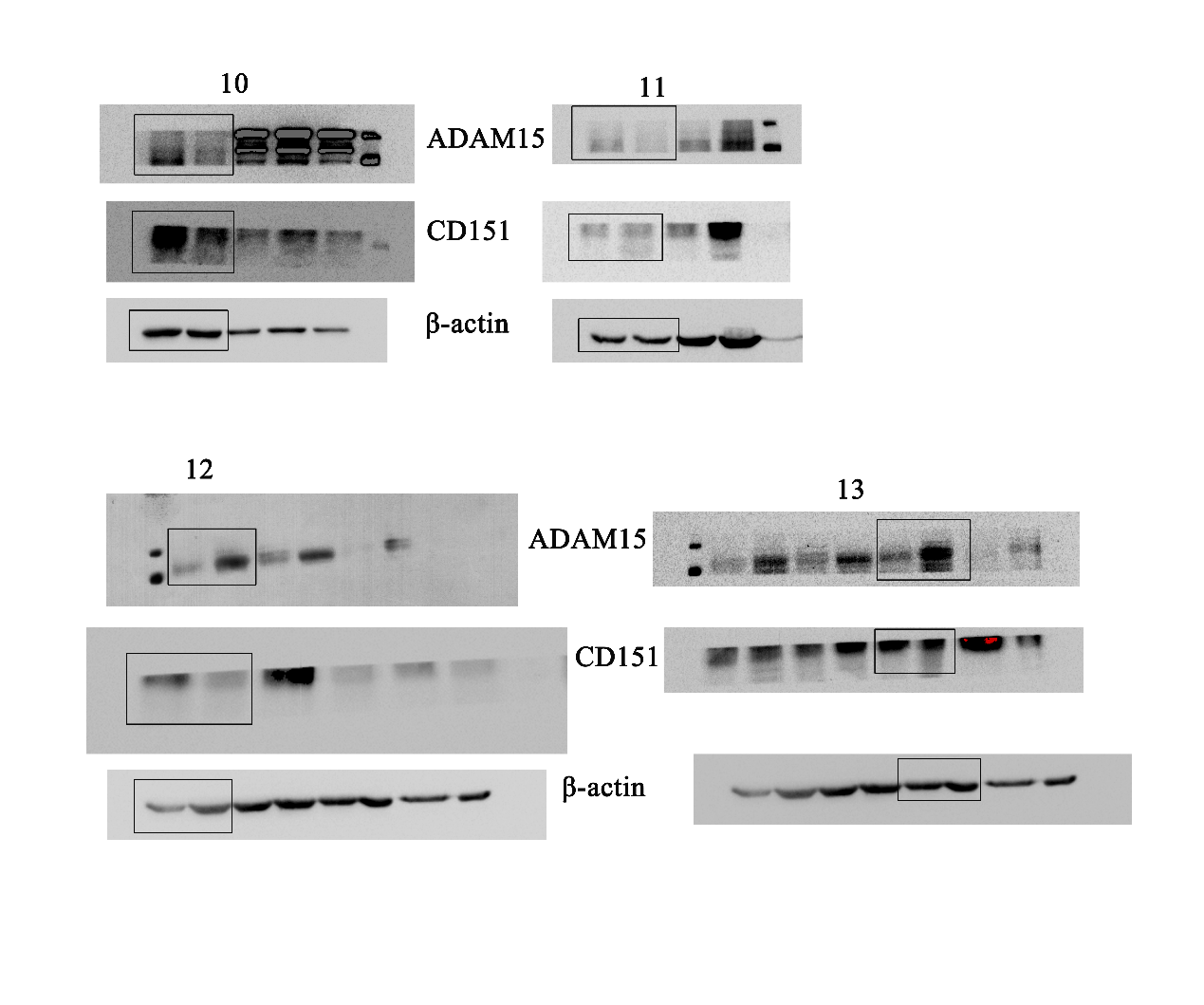


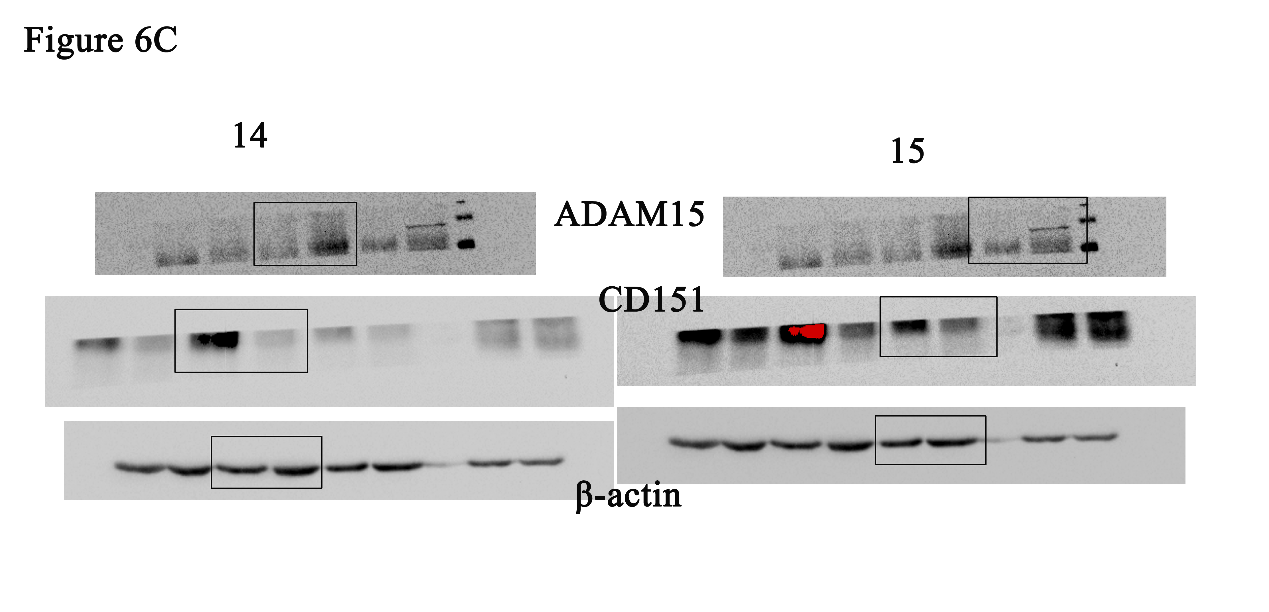


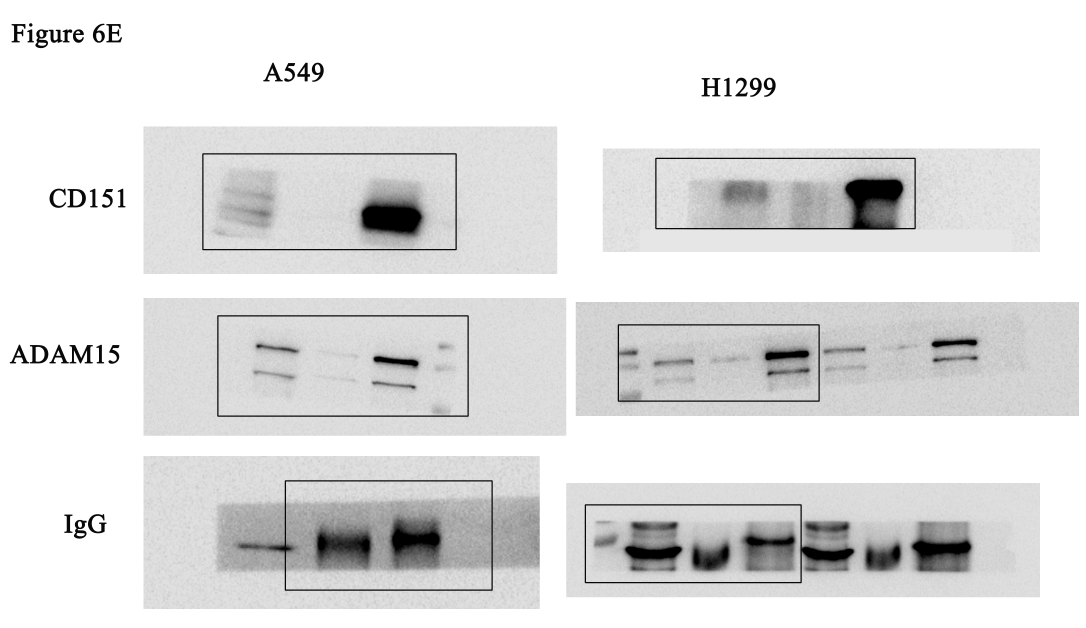


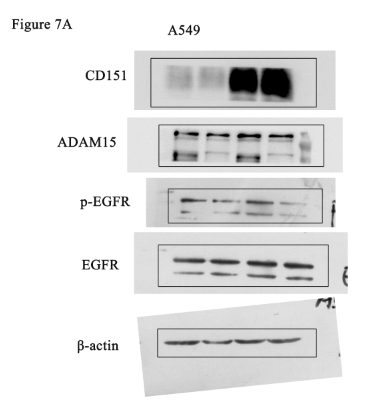

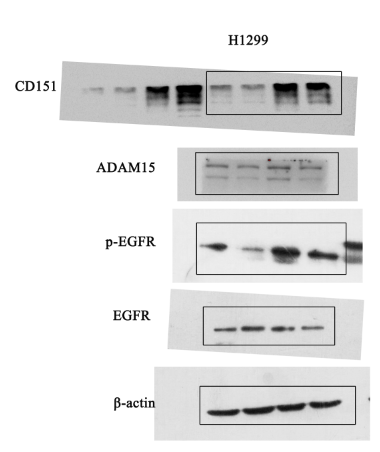


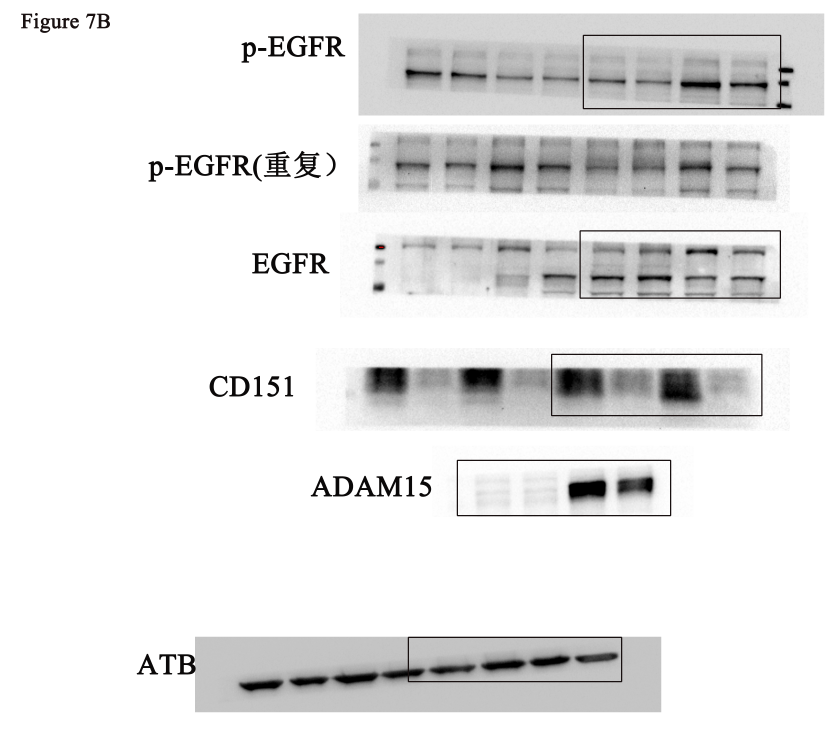


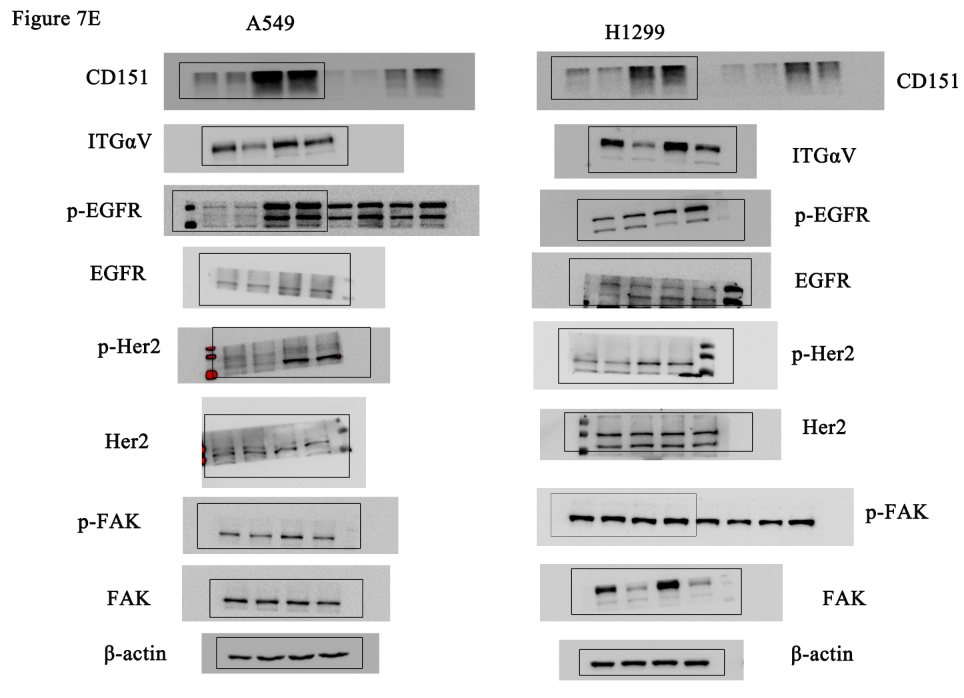


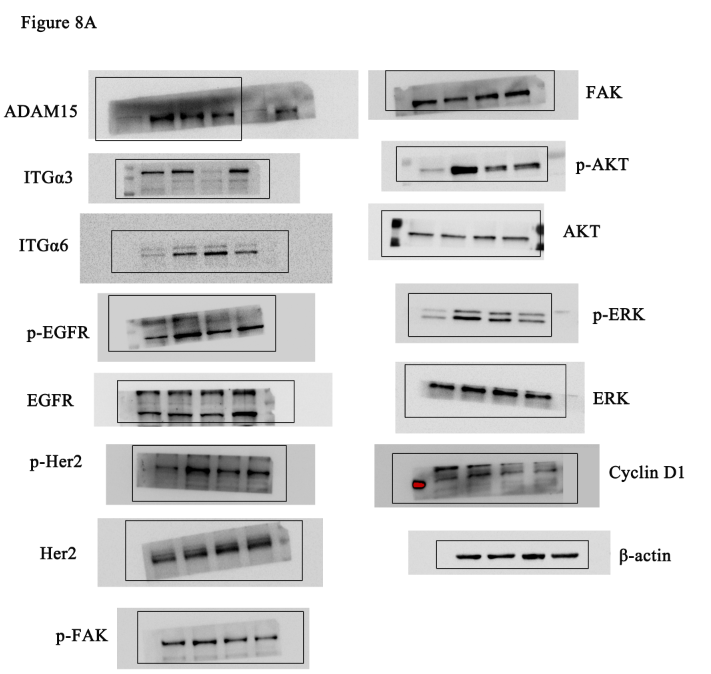


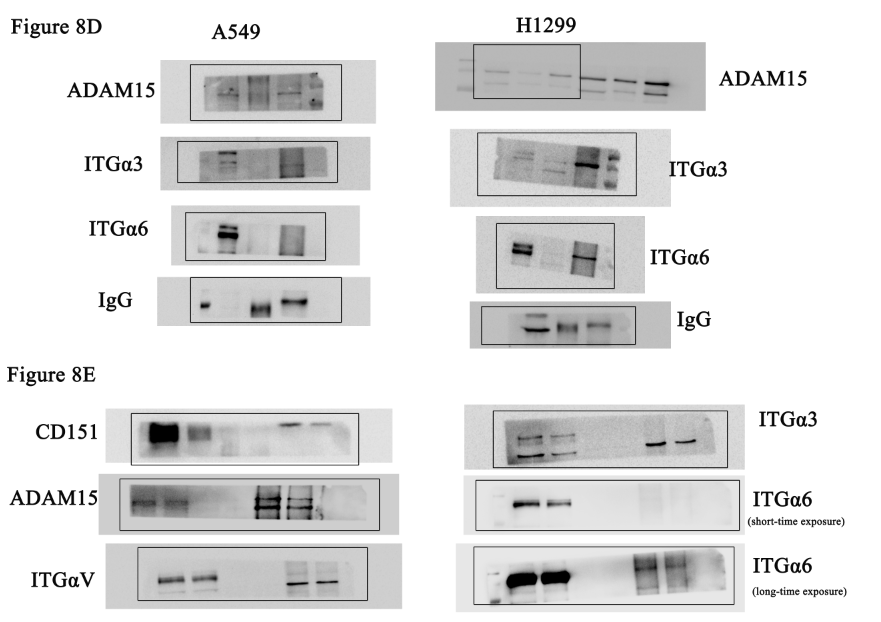


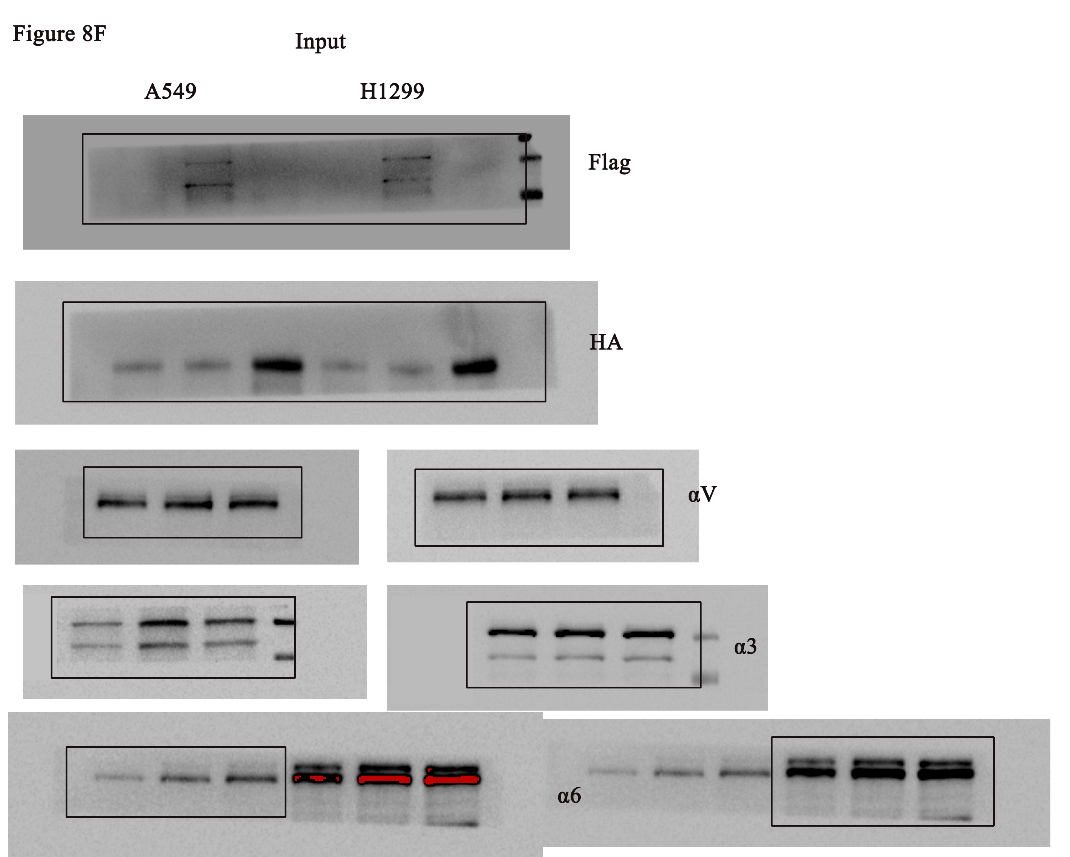


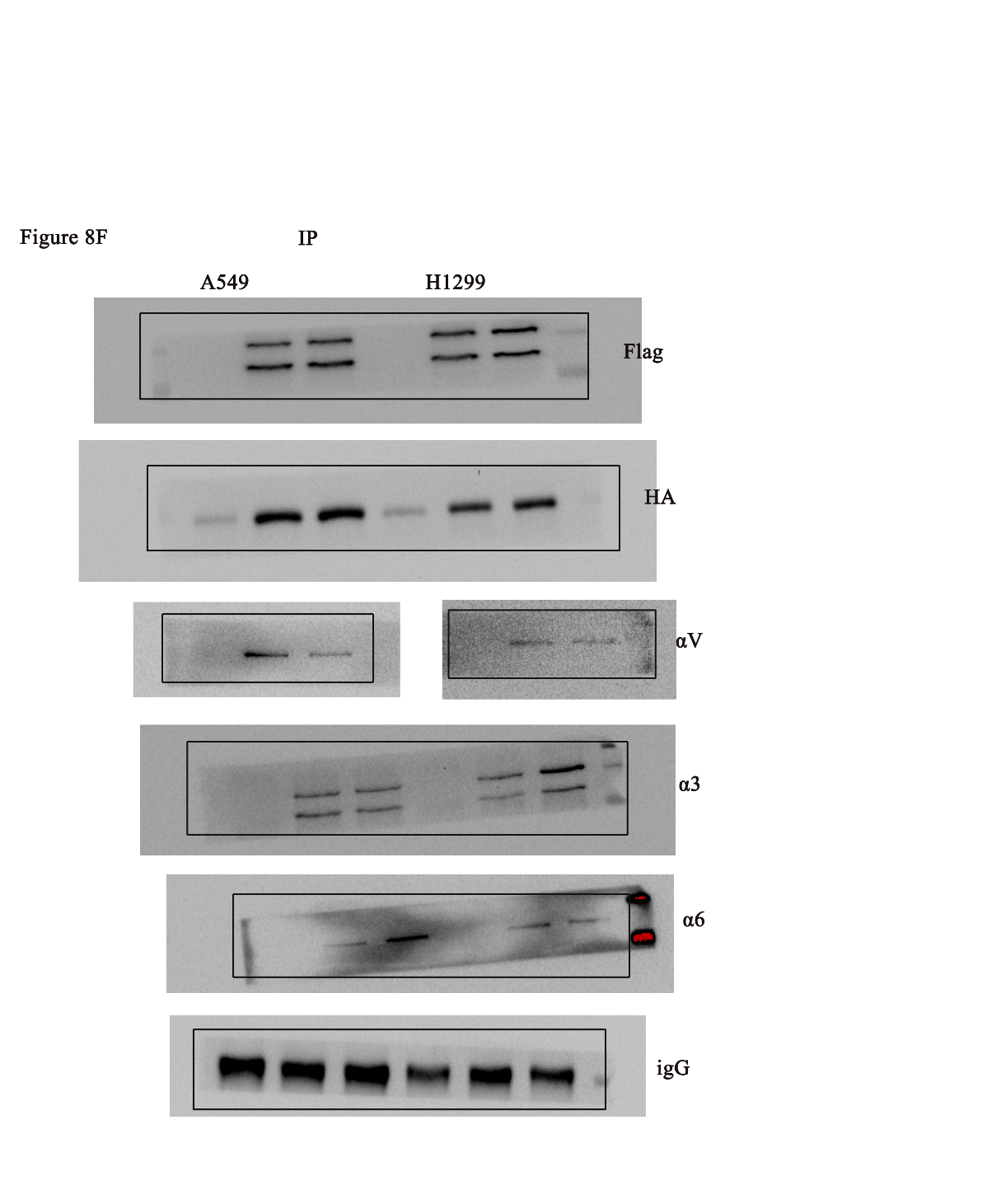


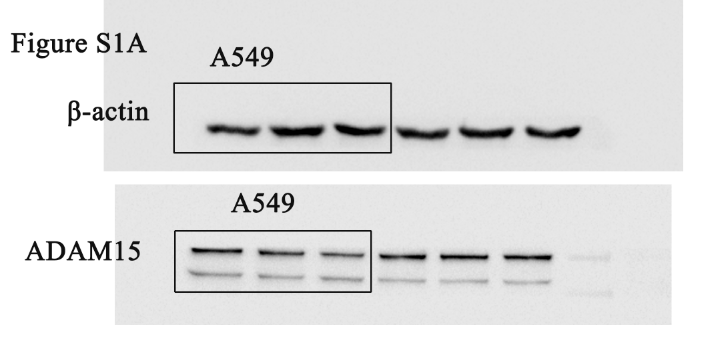


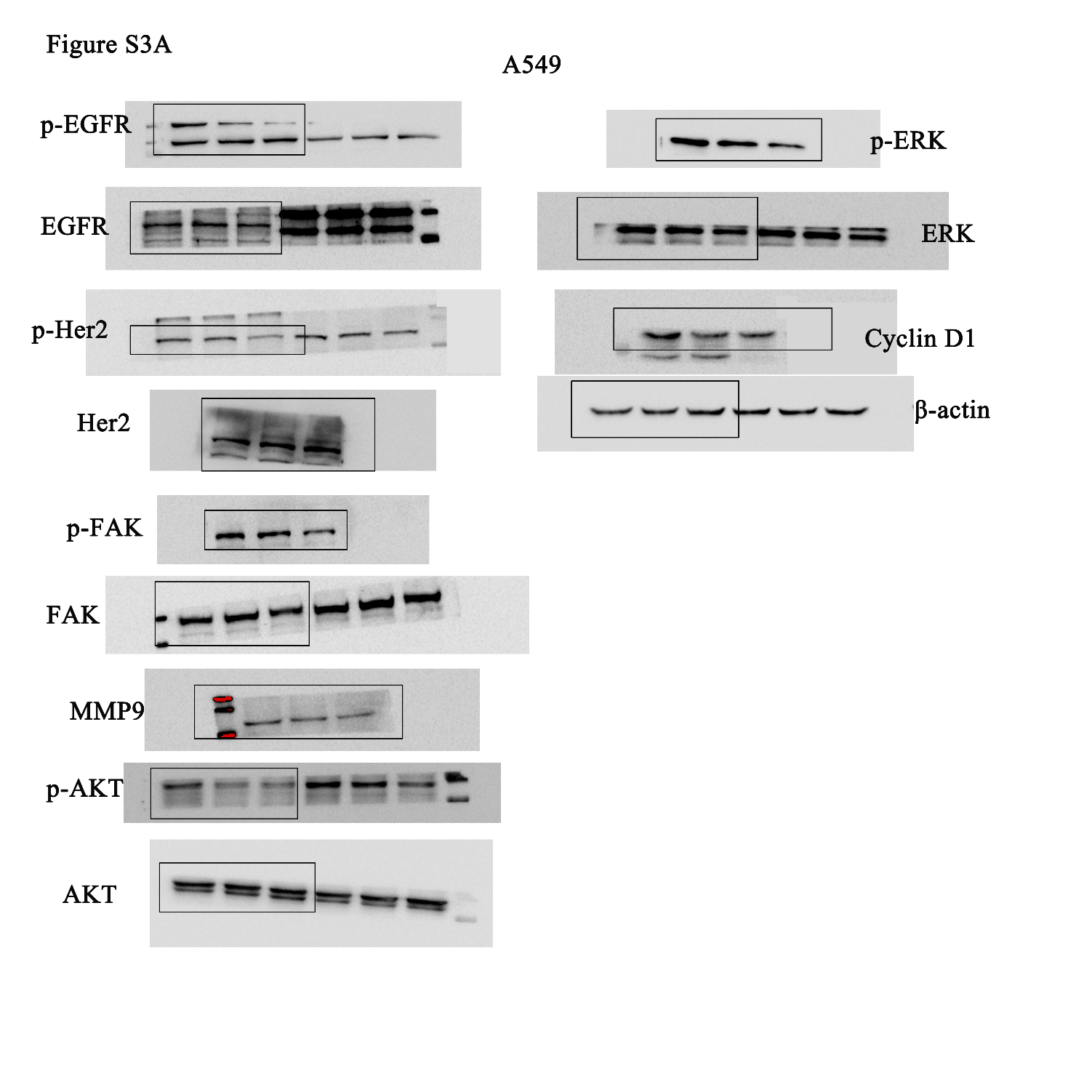


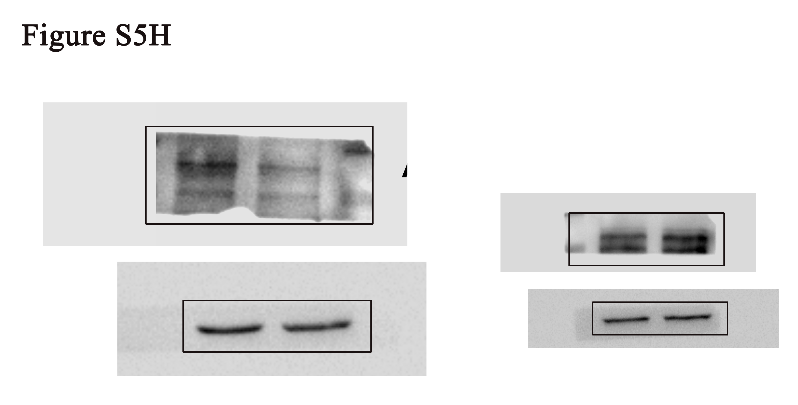


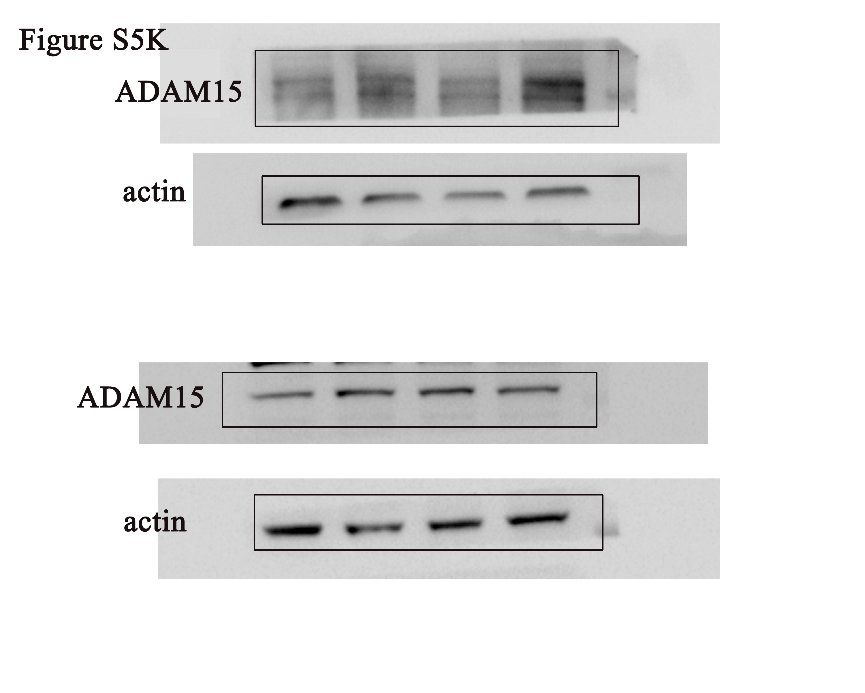


Raw data of the rebuttal letter

**Figure 2A**

A549


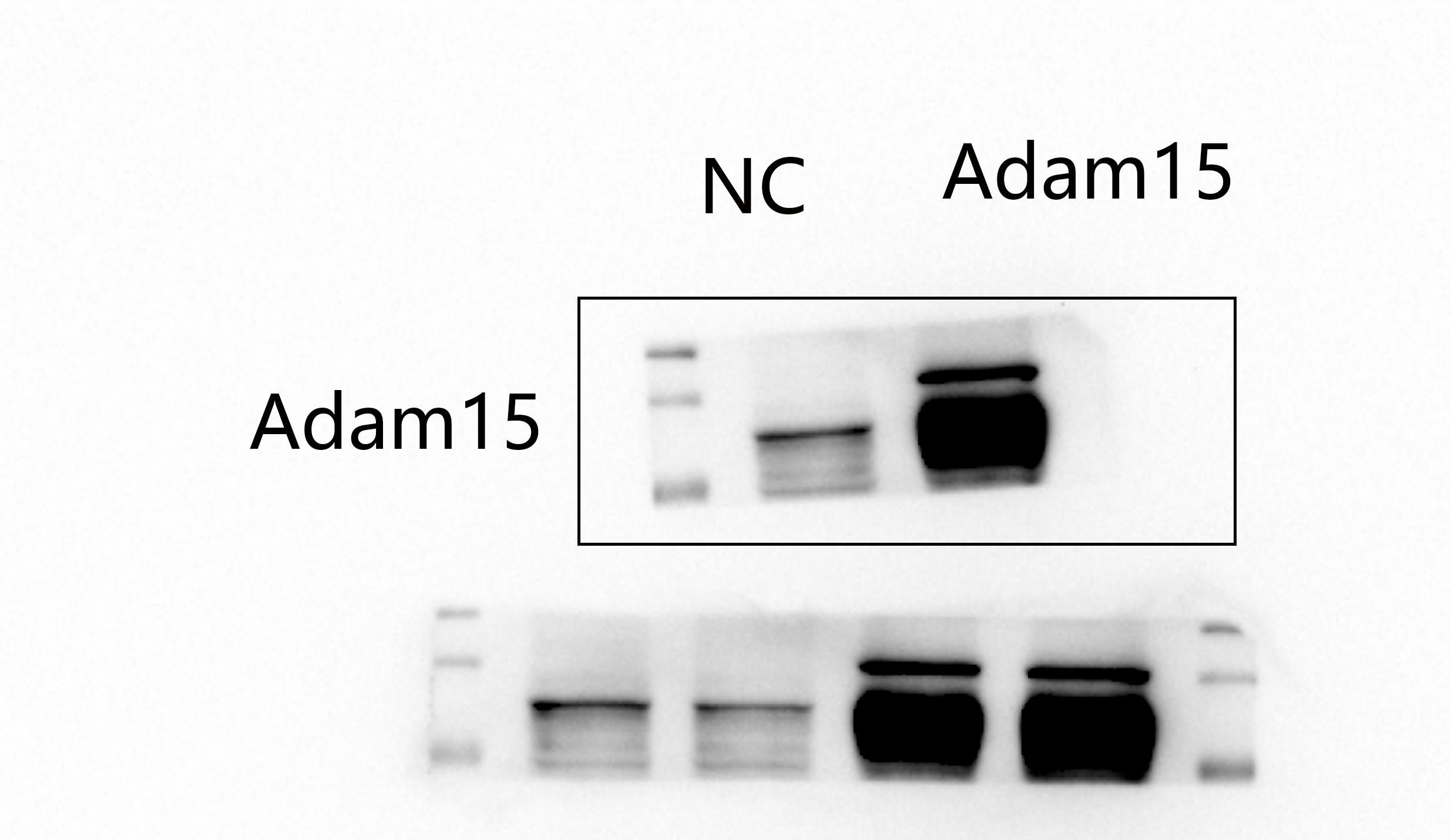

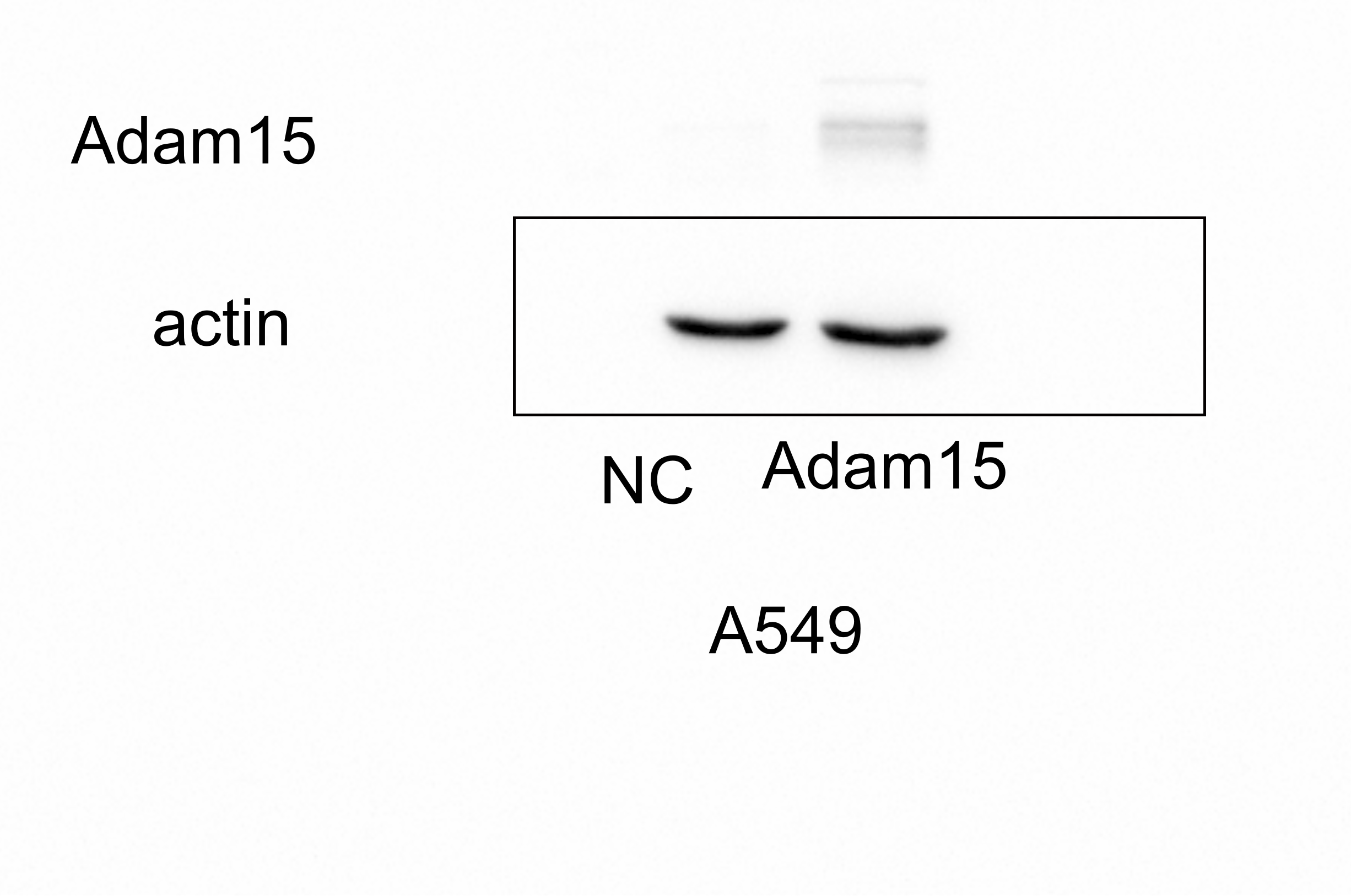


H1299


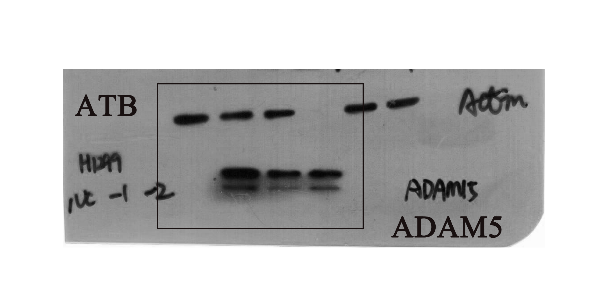


**Figure 4E**


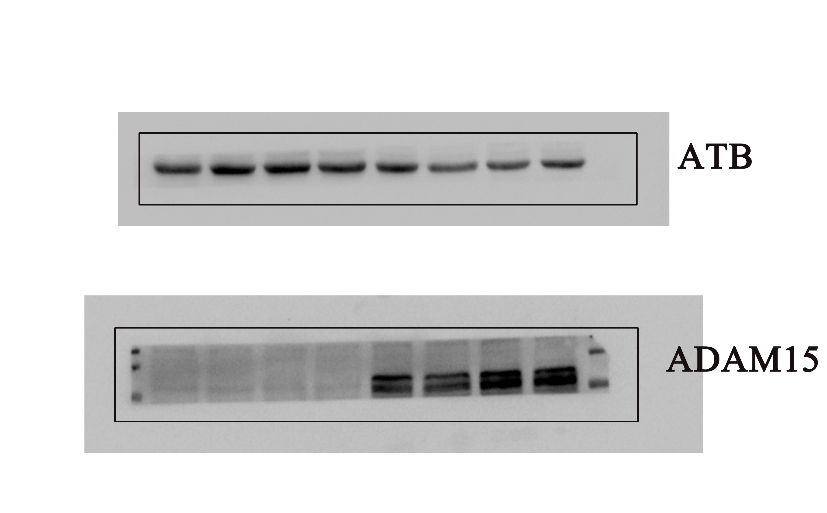


**Figure 4F**


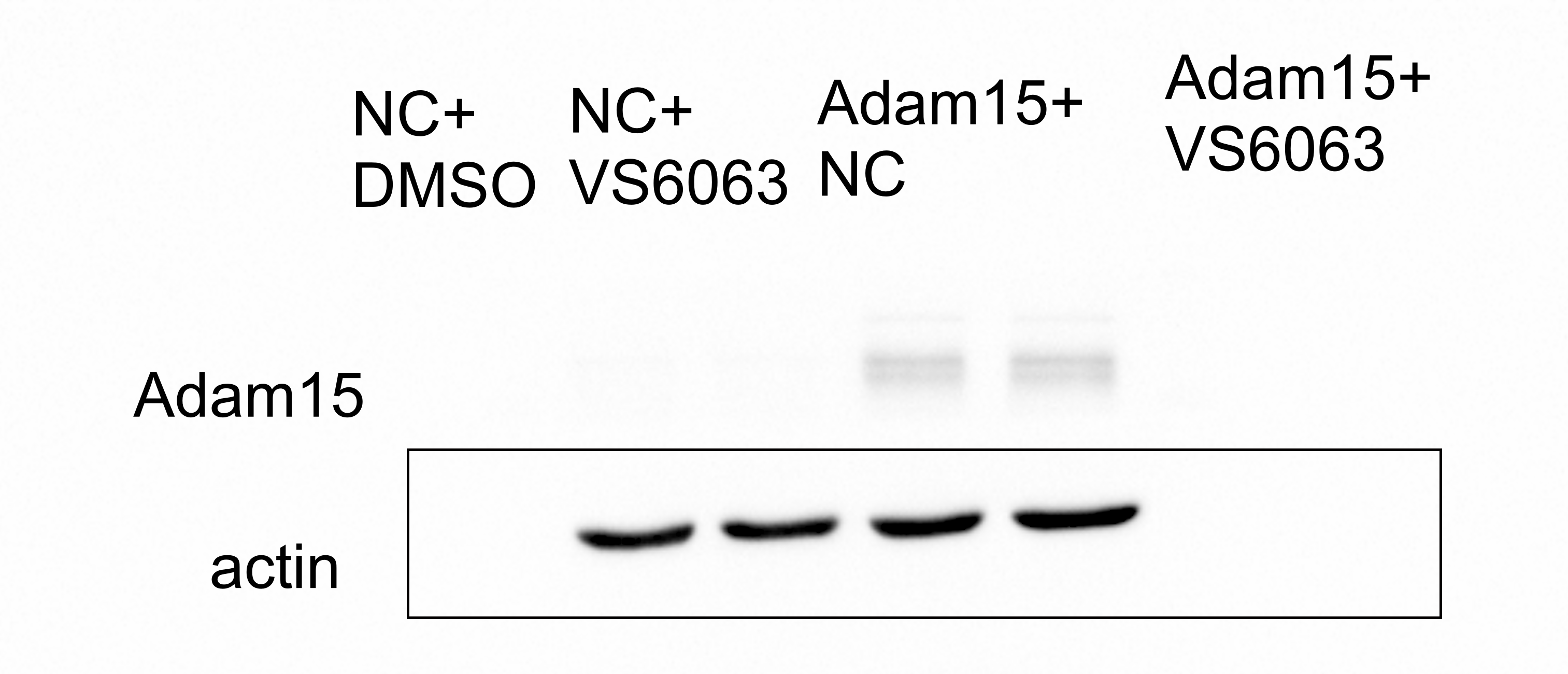

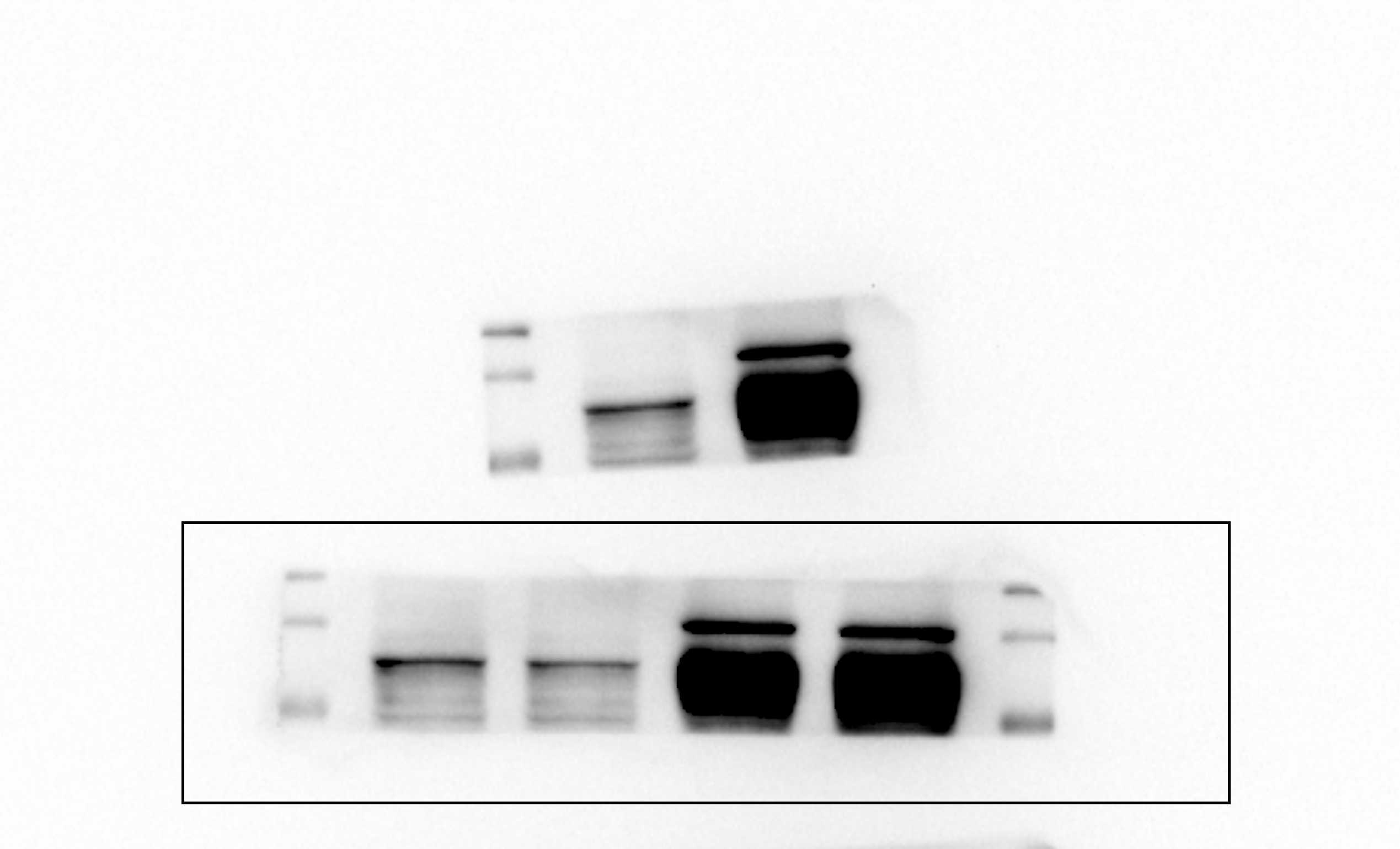


**Figure 5A**

**
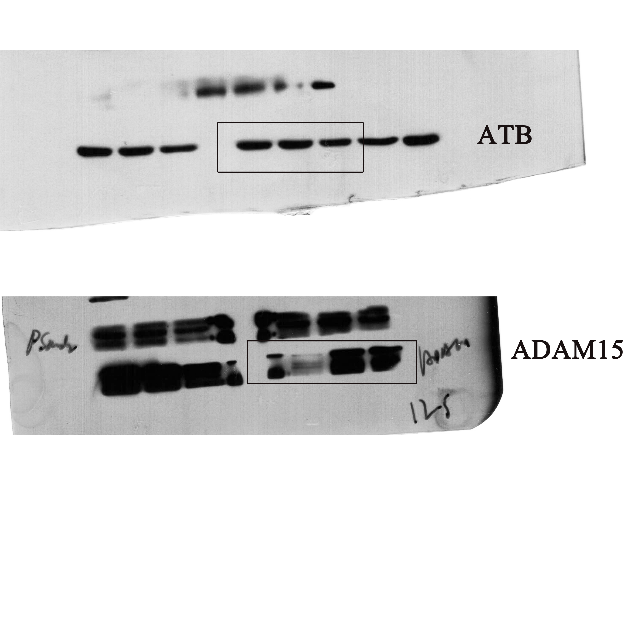
**

**Figure 7B, 8A**


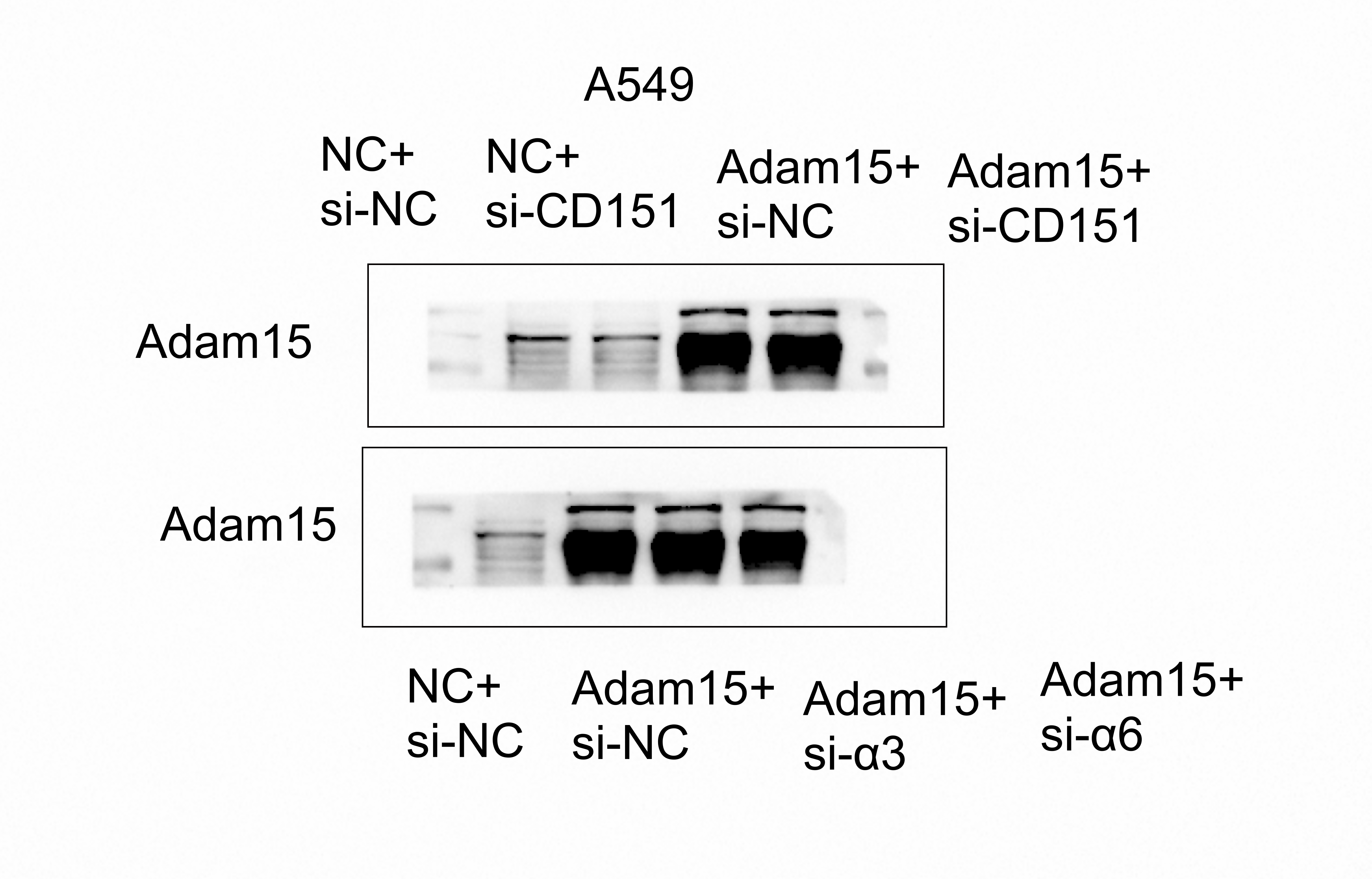

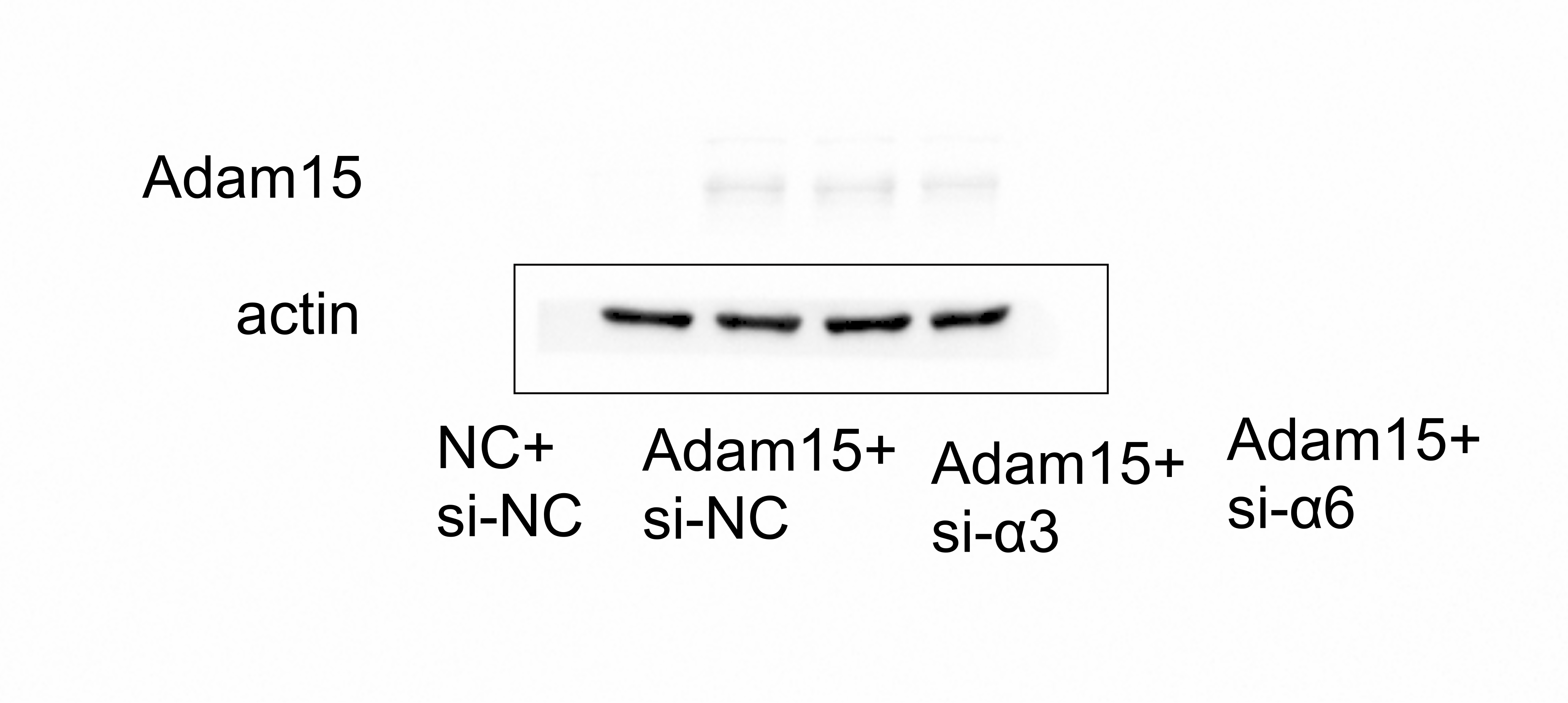

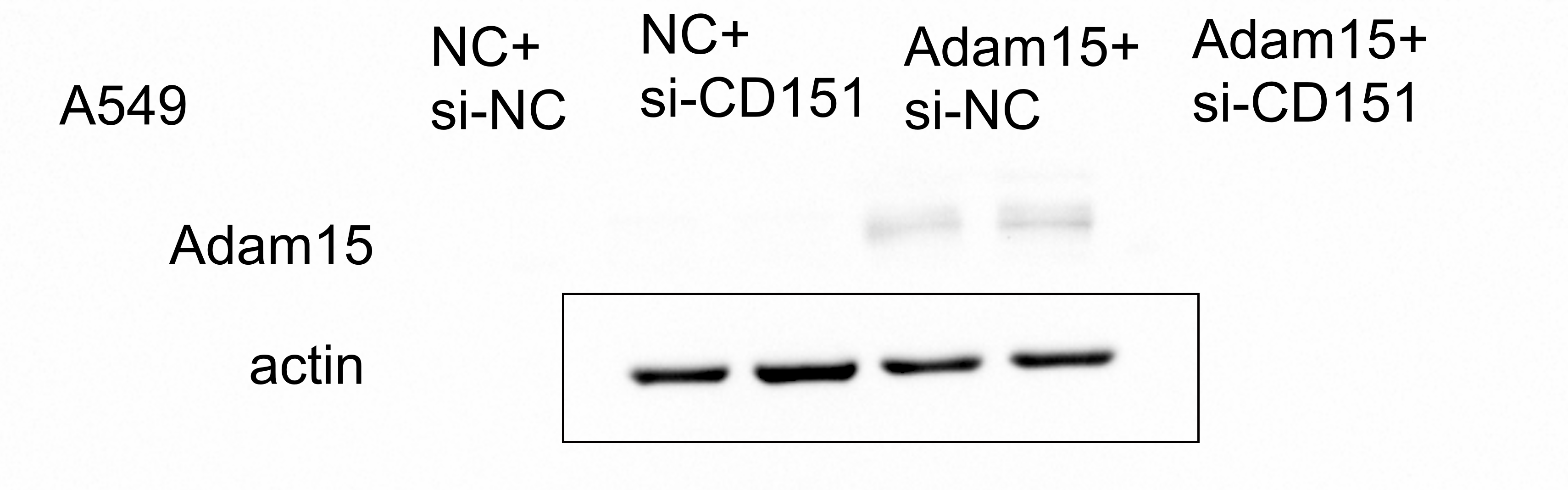

Supplement: Supplementary file 9 — Original Data File [file 41419_2022_4928_MOESM9_ESM.docx]
