# Supplementary material for: Integrin α3/α6 and αV are implicated in ADAM15-activated FAK and EGFR signalling pathway individually and promote non-small-cell lung cancer progression
Source: Cell Death Dis. 2022 May 21;13(5):486. doi: 10.1038/s41419-022-04928-0 (PMC9124216; doi:10.1038/s41419-022-04928-0)
Supplement: Supplementary file 6 — Table S1 [file 41419_2022_4928_MOESM6_ESM.docx]

​​

**Table S1 Demographic and clinical characteristics and levels of ADAM15 protein expression in NSCLC tissue**

| Characteristics | n=14 | ADAM15 protein expression  high(n=10) low(n=4) | | *P* value |
| --- | --- | --- | --- | --- |
| Age (years) |  |  |  |  |
| ≤60 | 4(28.6%) | 3 | 1 | 0.689 |
| >60 | 10(71.4%) | 7 | 3 |  |
| Gender |  |  |  |  |
| Male | 7(50.0%) | 5 | 5 | 0.72 |
| Female | 7(50.0%) | 2 | 2 |  |
| Histological features |  |  |  |  |
| Adenocarcinoma | 11(78.6%) | 8 | 3 | 0.494 |
| Squamous cell carcinoma | 1(7.1%) | 0 | 1 |  |
| Others | 2(14.3%) | 2 | 0 |  |
| Smoker |  |  |  |  |
| Yes | 5(35.7%) | 6 | 2 | 0.510 |
| No | 9(64.3%) | 3 | 2 |  |
| Clinical stage |  |  |  |  |
| I +II | 7(50%) | 6 | 1 | 0.280 |
| III + IV | 7(50%) | 4 | 3 |  |
| Distant metastasis |  |  |  |  |
| No | 13(92.9%) | 9 | 4 | 0.714 |
| Yes | 1(7.1) | 1 | 0 |  |
| Lymph node metastasis |  |  |  |  |
| No | 8(57.1%) | 7 | 1 | 0.175 |
| Yes | 6(42.9%) | 3 | 3 |  |

Data are presented as mean ± SD values. Kruskal-Wallis test for comparison between three or more groups.
