# Supplementary material for: Integrin α3/α6 and αV are implicated in ADAM15-activated FAK and EGFR signalling pathway individually and promote non-small-cell lung cancer progression
Source: Cell Death Dis. 2022 May 21;13(5):486. doi: 10.1038/s41419-022-04928-0 (PMC9124216; doi:10.1038/s41419-022-04928-0)
Supplement: Supplementary file 7 — Table S2 [file 41419_2022_4928_MOESM7_ESM.docx]

**Table S1 Clinical characteristics of NSCLC patients and the level of ADAM15 and CD151 protein expression in tumor tissue specimens**

| ID | Gender | Age | Histology | T status | N status | Clinical stage | ADAM15 protein Expression | CD151 protein Expression |
| --- | --- | --- | --- | --- | --- | --- | --- | --- |
| Patient 1 | male | 71 years | Adenocarcinoma | T1 | N1 | Ⅱb | High | / |
| Patient 2 | male | 68 years | Adenosquamous carcinoma | T2 | N0 | Ⅱa | High | / |
| Patient 3 | male | 62 years | Squamous carcinoma | T4 | N1 | Ⅲa | High | High |
| Patient 4 | male | 61 years | Squamous carcinoma | T2 | N0 | Ⅰb | High | High |
| Patient 5 | male | 68 years | Adenocarcinoma | T1 | N0 | Ⅰb | High | High |
| Patient 6 | female | 69 years | Adenocarcinoma | T2 | N0 | Ⅱa | Low | High |
| Patient 7 | female | 59 years | Adenocarcinoma | T2 | N0 | Ⅰb | High | High |
| Patient 8 | male | 65 years | Adenosquamous carcinoma | T4 | N0 | Ⅲa | Low | High |
