# Supplementary material for: Integrin α3/α6 and αV are implicated in ADAM15-activated FAK and EGFR signalling pathway individually and promote non-small-cell lung cancer progression
Source: Cell Death Dis. 2022 May 21;13(5):486. doi: 10.1038/s41419-022-04928-0 (PMC9124216; doi:10.1038/s41419-022-04928-0)
Supplement: Supplementary file 8 — Supplementary materials. [file 41419_2022_4928_MOESM8_ESM.docx]

**SUPPLEMENTARY FIGURE LEGENDS**

**Fig S1. ADAM15 affects NSCLC cell proliferation, migration and invasion in ADAM15-knockdown A549 cell lines.**

(A) ADAM15 mRNA and protein levels were detected in ADAM15-knockdown A549 cell lines. (B) CCK-8 assay of cell proliferation in A549 cell lines. (C) A wound healing assay was performed to evaluate the effect of ADAM15 in A549 cell lines. (D) Images of the Transwell assay results for cell migration and invasion in A549 cell lines. Bars represent the mean ± SEM from three independent experiments. *P < 0.05; **P< 0.01; ***P < 0.001.

**Fig S2. Flow cytometry analysis of the NSCLC cell lines.**

**Fig S3. ADAM15 affects the EGFR/Her2-FAK signalling pathways in ADAM15-knockdown A549 cell lines.**

(A) The expression levels of various proteins were measured by western blot analysis in ADAM15-knockdown A549 cell lines. β-actin was used as a loading control. *P < 0.05; **P< 0.01; ***P < 0.001. (B) Data from the CCLE database and analysed by GSEA software.

**Fig S4. NSCLC samples were immunostained with anti-ADAM15 antibody and anti-CD151 antibody. Representative samples are shown.**

**Fig S5. MiR-204-5p targets ADAM15 to inhibit cell proliferation.**

(A) Multiple databases (miRWalk, Targetscan, and miRactDB) were used to predict the regulatory miRNA of ADAM15. (B) ADAM15 was negatively related to miR-204-5p. (C) Prediction of the possible binding sites of ADAM15 and miR-204-5p by TargetScan. (D) TCGA database was analysed to compare the miR-204-5p expression levels in 45 normal and 483 lung cancer tissues (https://portal.gdc.cancer.gov/). (E) qRT-PCR analysis of miR-204-5p levels in different non-small-cell lung cancer cells. (F) Kaplan-Meier analysis of overall survival for miR-204-5p expression in 504 adenocarcinoma samples. Kaplan–Meier plots were generated using Kaplan–Meier Plotter (http://www.kmplot.com). (G) The dual-fluorescence reporter assay detected the change in relative dual fluorescein activity after cotransfection of miR-NC or miR-204-5p mimics and ADAM15 wild-type or mutant plasmid. (H, I) qRT-PCR detected changes in miR-204-5p and ADAM15 mRNA expression after transfection with miR-204-5p mimics or inhibitors. (J) Western blot analysis of the change in ADAM15 protein expression after transfection with miR-204-5p mimics or inhibitors. (K, M) CCK-8 and clonogenic assay of cell proliferation in A549 cell lines. (L, N) qRT-PCR and western blot detected the change in ADAM15 mRNA expression after transfection with miR-let-7b, miR-let-7c and miR-3174 mimics. β-actin was used as a loading control. *P < 0.05; **P< 0.01; ***P < 0.001.

**Fig S6. Raw data of Fig 6C.**

**Table S1. Demographic and clinical characteristics and levels of ADAM15 protein expression in NSCLC tissue.**

**Table S2. Clinical characteristics of NSCLC patients and the level of ADAM15 and CD151 protein expression in tumour tissue specimens.**

**SUPPLEMENTARY MATERIALS AND METHODS**

**2.3 Immunohistochemical assay**

Adjacent sections of serial paraffin sections were incubated with anti-CD151 (Santa Cruz, CA, USA, 1:60 dilution in 5% BSA in PBS) and anti-ADAM15 antibodies (Abcam, London, UK, 1:200 dilution in 5% BSA in phosphate-buffered saline (PBS)) at 4°C overnight and then incubated with the corresponding biotinylated secondary antibodies. The reactions were developed using the DAB Kit (BD Biosciences, San Jose, CA, USA), and the slides were counterstained with haematoxylin. Briefly, the proportion score was graded as follows: staining in 0% of the cells examined was counted as 0; 0.01%–25% was counted as 1; 25.01%–50% was counted as 2; 50.01%–75% was counted as 3; and 75%-100% was counted as 4. The staining intensity was graded as follows: 0, no signal; 1, weak; 2, moderate; and 3, strong. The histological score for each section was computed using the formula: histological score = proportion score × intensity score. A total score in the range of 0–12 was calculated and graded as follows: negative (-, score: 0), weak (+, score: 1–4), moderate (++, score: 5–8) or strong (+++, score: 9–12). Scores of " - " and "+" were considered to indicate low expression levels, whereas scores of "++" and "+++" were considered to indicate high expression levels.

**2.4 RNA extraction and quantitative real-time PCR analysis**

We used reverse transcriptase M-MLV (TaKaRa, Osaka, Japan) to synthesize cDNA. Primer sequences for mRNA detection were as follows: ADAM15: 5′-AGCCTCAAAAAGGT GCTTCA-3′ (forward), 5′-TGGTAGCAGCAGTTCTC-3′ (reverse); β-actin: 5′-CACAGAGC CTCGCCTTTGCC-3′ (forward), 5′-ACCCATGCCCACCATCACG -3′ (reverse). We used SYBR Premix Ex Taq^TM^ (TaKaRa, Osaka, Japan) and an ABI Step One Plus Real-Time PCR system (Applied Biosystems, Foster City, CA, USA) to perform real-time PCR analysis.

**2.5 RNA interference**

The target sequences of the siRNAs were as follows: ADAM15-1 siRNA: 5′-CCCAGCUGUCACCCUCGAATT-3′; ADAM15-2 siRNA: 5′-GAUCUACUCUGGGAGAC AATT-3′; ITGαV siRNA: 5′-GGUCCAAGUUCAUUCAGCAAGGCAA-3′; ITGα3 siRNA: 5′-UUACAGAGACUUUGACCGATT-3′; ITGα6 siRNA: 5′-CAAACAGCUCAUAUUGAU TT-3′.

**2.7** **Construction of Flag-tagged ADAM15 and HA-tagged CD151 expression vectors**

Primer sequences are as follows: ADAM15: 5′ - AAGCTGGCTAGCATGCGGCTGGCGCTG-3′ (forward), 5′ - GTAGTCACCGGTGAGGTAGAGCGAGGACACT-3′ (reverse); CD151: 5′ - AGACCCAAGCTGGCTAGCGCCACCATGGGTGAGTTCAA -3′ (forward), 5′ - AACA TCGTATGGGTAACCGGTGTAGTGCTCCAGCTTGAGACTCCTGTA-3′ (reverse).

**2.9 Co-immunoprecipitation (co-IP) assay**

We seeded the cells in a 100 mm plate until the density reached 90%-100%, and then we lysed the cells with 1 ml of modified RIPA buffer (Cell Signaling Technology, Danvers, MA, USA) containing protease and phosphatase inhibitor cocktail (Sigma-Aldrich, St. Louis, MO, USA) for 30 min after washing twice with cold phosphate-buffered saline (PBS). Cell lysates were collected by centrifugation at 10000×g at 4°C for 30 min. Then, the supernatants were transferred to a new Eppendorf tube, 1 µg of IgG or the antibody against the target gene was added to each tube and incubated at 4°C for 24 h with rotation. Then, 50 µl of protein G bead slurry was added to the supernatants, which were incubated at 4°C for 24 h with rotation. The beads were washed three times with RIPA buffer and then boiled in 2× SDS protein loading buffer for 5 min. Samples (20 μl) were loaded on SDS-PAGE gels for western blot analysis.

**2.10 Dual-luciferase reporter assay**

TargetScan software was used to predict the possible binding sites of miR-204-5p and the 3'-UTR segment of the ADAM15 gene. The fragment containing the predicted binding site was combined with the 3' end of the dual-luciferase reporter molecule to form a wild-type plasmid. The fragment containing the predicted binding site was mutated and combined with the 3' end of the dual-luciferase reporter molecule to form a mutant plasmid, which was connected to the psiCHECK2 vector. A549 cells were seeded in 24-well plates and cotransfected with wild-type or mutant plasmids along with either miR-NC or miR-204-5p mimic using Lipofectamine 2000 (Invitrogen, Carlsbad, CA, USA), and a blank plasmid was constructed as a control. After incubation for 48 h, the cell lysates were harvested. The luciferase activity was assessed by a Dual-Luciferase Reporter Assay Kit (Promega) and then standardized to the Renilla luciferase activity. Each experiment was performed independently in triplicate.
